# Work-family conflict and mental health: A systematic review and meta-analysis

**DOI:** 10.1371/journal.pmed.1005162

**Published:** 2026-07-17

**Authors:** Dongyu Liu, Yanyan Ni, Tiffany S. W. Ma, Xiaoyan Fan, Yongle Zhan, Ivan H. L. Lam, Matthew C. L. Lui, Tarani Chandola, Ichiro Kawachi, Pim Cuijpers, Michael Y. Ni

**Affiliations:** 1 School of Public Health, LKS Faculty of Medicine, The University of Hong Kong, Hong Kong, The Hong Kong Special Administrative Region of the People’s Republic of China; 2 Department of Surgery, School of Clinical Medicine, LKS Faculty of Medicine, The University of Hong Kong, Hong Kong, The Hong Kong Special Administrative Region of the People’s Republic of China; 3 LKS Faculty of Medicine, The University of Hong Kong, Hong Kong, The Hong Kong Special Administrative Region of the People’s Republic of China; 4 Department of Sociology, The University of Hong Kong, Hong Kong, The Hong Kong Special Administrative Region of the People’s Republic of China; 5 Harvard T.H. Chan School of Public Health, Harvard University, Boston, Massachusetts, United States of America; 6 Department of Clinical, Neuro and Developmental Psychology, Amsterdam Public Health research institute, Vrije Universiteit Amsterdam, Amsterdam, The Netherlands; 7 Urban Systems Institute, The University of Hong Kong, Hong Kong, The Hong Kong Special Administrative Region of the People’s Republic of China; National Institute of Mental Health and Neuro Sciences, INDIA

## Abstract

**Background:**

Work-family conflict, defined as a type of inter-role conflict where demands from work and family roles are incompatible, is a global public health concern. Cross-sectional studies consistently demonstrate associations between work-family conflict and adverse mental health outcomes. However, longitudinal evidence remains mixed, highlighting the need for a comprehensive synthesis of available data to better understand its long-term impacts.

**Methods and findings:**

We performed a systematic review and meta-analysis of longitudinal studies of associations between work-family conflict and mental health outcomes, involving 112,714 participants and 52 studies in the meta-analysis. The review was prospectively registered in PROSPERO (CRD42022325540). We searched PubMed, Web of Science, Scopus, and PsycINFO for English-language articles and SinoMed for Chinese-language articles in February–March 2026. We further performed backward citation searches by screening the reference lists of previously published reviews to ensure the comprehensive inclusion of all eligible studies. Eligible studies were original longitudinal research focusing on adult workers, examining work-to-family and/or family-to-work conflict as exposures related to mental health outcomes. Risk of bias was assessed using the Newcastle–Ottawa Scale. Random-effects meta-analyses were conducted for outcomes with three or more comparable studies, converting effect sizes to standardised regression coefficients (*β*), indicating standard deviation (SD) differences in mental health per 1-SD increase in work-family conflict. We synthesised effect sizes with the most comprehensive confounding adjustments when possible, including sociodemographic, work/family-related factors, and baseline mental health. Work-to-family conflict was associated with depressive symptoms (β = 0.10, 95% confidence interval [0.06, 0.15]; *p* < 0.001), burnout (β = 0.11, 95% CI [0.01, 0.21]; *p* = 0.040), and general mental distress (β = 0.13, 95% CI [0.03, 0.24]; *p* = 0.005), independent of baseline mental health and other confounders. Significant associations were also found for family-to-work conflict with depressive symptoms (β = 0.09, 95% CI [0.04, 0.15]; *p* = 0.006) and burnout (β = 0.08, 95% CI [0.03, 0.13]; *p* = 0.013) in studies with similar adjustments. No significant differences emerged based on gender, follow-up length, and geographical location. While evidence of publication bias was identified, sensitivity analyses employing a trim-and-fill method to adjust for this bias yielded broadly similar pooled estimates. A causal relationship cannot be fully established as all identified studies were observational.

**Conclusions:**

These findings provide evidence of the longitudinal association between work-family conflicts and mental health outcomes, underscoring the need for context-specific policies in promoting work-family balance and mental health.

## Author summary

**Why was this study done?:**

- The modern workforce faces competing demands from work and family responsibilities, leading to work-family conflict when job demands disrupt family life and/or family responsibilities hinder work performance.
- Previous reviews showed a link between work-family conflict and poorer mental health. However, most evidence came from cross-sectional studies, making it unclear whether conflict leads to mental health issues or vice versa.
- Previous reviews have mixed cross-sectional and longitudinal studies and combined studies with different confounding adjustment conditions, which increases the risk of biased findings.
- It is also uncertain whether the mental health impact of work-family conflict could be moderated by factors such as follow-up length, gender, and geographic location.

**What did the researchers do and find?:**

- We conducted a systematic review of 82 longitudinal studies and meta-analysed 52 studies to examine the longitudinal associations between work-family conflict and mental health outcomes.
- We found associations of work-to-family conflict with subsequent depressive symptoms, burnout, and general mental distress in studies that adjusted for sociodemographic, work-related factors, and baseline mental health. Similar associations were identified for family-to-work conflict with depressive symptoms and burnout.
- The associations were broadly similar across men and women, shorter and longer follow-up periods, and for the geographical settings available for comparison.

**What do these findings mean?:**

- Work-family conflict is an important and modifiable contributor to workers’ mental health, which should be considered in workplace health promotion and policy.
- Given the high prevalence of work-family conflicts globally, addressing this issue requires changing social norms to promote more gender equality (e.g., in the household division of labour) and implementing social policies that support working families, including parental leave reforms, expanded access to high-quality care for children and older adults, flexible working arrangements, and clearer work-home boundaries.
- All identified studies rely on observational designs and predominantly originate from high-income Western settings and Chinese populations. More high-quality studies across a wider range of cultural and social contexts are needed. Future quasi-experimental studies with matched exposed and unexposed groups could strengthen causal evidence.

## Abstract 3

Dongyu Liu and colleagues perform a systematic review and meta-analysis of longitudinal studies of work-family conflict to examine associations with mental health impacts.

## Introduction

A major challenge faced by the global workforce is work-family conflict, which is a type of inter-role conflict where demands from work and family roles are incompatible [1–4]. This conflict could manifest as: 1) work-to-family conflict, where work demands negatively impact family life, and 2) family-to-work conflict, where family responsibilities interfere with work performance [3]. Representative surveys highlighted work-family conflict as a widespread issue, for instance, affecting up to a third of European working parents [4], driven by both workplace and family factors. Workplace factors include unpredictable work schedules (“Just in Time” scheduling) and blurred boundaries due to the “always-on” culture, which increases work intrusion into family life [5–8]. Simultaneously, the rise in single-parent households and care-taking responsibilities for children and older people intensifies family demands that compete with paid work [5–9]*.*

The prevalence and experience of work-family conflict can vary across regions, driven by differences in labour-market conditions, family policies, caregiving systems, and cultural norms. For instance, in the US, the Family and Medical Leave Act provides only 12 weeks of unpaid leave, and covers only 56%–60% of the workforce [10–12]. The combined challenges from limited parental leave, insufficient access to affordable and high-quality childcare and care for older people, long commuting hours, long working hours, and a lack of supportive work-family policies exacerbate the problem in many regions [5,10,13]. Notably, one-fifth of the global workforce and half of the workforce in Asian regions are working long hours, defined as more than 48 hours per week [5,14]. Workers in Asia also face prolonged commuting time, with average daily travel reaching 53 min in Chinese metropolises and 58 min in South Korea [15,16]. Additionally, gender norms that shape the division of unpaid household and care responsibilities may further intensify work-family conflicts, particularly in dual-earner households. Evidence indicates that women continue to shoulder a greater share of unpaid domestic and care work than men, with this imbalance being especially pronounced in Asia [17].

Work and family represent the two most significant time commitments and priorities in most individuals’ lives [18,19]. As illustrated by the Work-Home Resources model [20], demands in the work and home domains draw on shared personal resources such as time, energy, and mood. Demands in one domain can thus deplete these resources and hinder functioning in the other. The resulting conflicts between these two domains can generate substantial stress and potentially lead to adverse mental health consequences, as suggested by cross-sectional studies [21,22]. If verified, this would have a major public health implication given the high prevalence of work-family conflict around the world [2,4,23]. Current research on the association between work-family conflict and mental health is predominantly based on observational survey studies. Such observational studies require rigorous adjustment for confounders and prior mental health to facilitate causal inferences [24,25]. Work-family conflict is influenced not only by sociodemographic characteristics but also by other work- and family-related stressors [23,26,27], all of which have established links to mental health outcomes [28,29]. Work-family conflict is derived from high demands from work and home domains [20]. These demands, such as long working hours and high childcare burdens, can also act as stressors that elevate risks of poor mental health [23,26–29], thereby biasing estimated associations away from the null if insufficiently adjusted for. Accordingly, synthesising evidence from studies that employ comprehensive confounding adjustment is essential for generating more reliable conclusions. Nevertheless, existing reviews and meta-analyses in work-family conflict predominantly neglected confounding adjustment conditions when synthesising evidence by pooling effect estimates from models with heterogeneous covariate adjustment conditions [21,30–33]. Mixing estimates that differ in adjustment conditions hampers comparability across estimates, while the under-adjusted estimates could bias the pooled effect due to common causes between work-family conflict and mental health [23,26–29].

Another major limitation of existing meta-analyses is mixing cross-sectional and longitudinal studies [21,30–32]. Cross-sectional studies are prone to reverse causality, and thus cannot clearly identify determinants of mental health [34]. One meta-analysis of work-related, family-related, and domain-general outcomes pooled results from cross-sectional and longitudinal studies without stratifying by study design [21]. Another cross-national meta-analysis attempted to provide a synthesised effect size from longitudinal studies, but this study only explored life satisfaction, not general mental health or specific mental health symptoms [30]. A mega-meta path study also identified no synthesised longitudinal effect size for the association between work-family conflict and mental health from previous meta-analysis studies [31]. Moreover, many previous reviews are limited in scope, concentrating on one specific outcome [32,33], certain countries [1,35], or high-risk occupations [32]. To date, no meta-analysis has synthesised longitudinal evidence with comprehensive confounding adjustment, encompassing working populations across occupations and countries, while addressing a broad spectrum of mental health outcomes in the context of work-family conflict.

Moreover, it remains unclear whether the impact of work-family conflict could be moderated by factors such as follow-up length, gender, and geographic location [36–40]. Longitudinal studies exhibit inconsistencies [1], with some reporting null associations when follow-up intervals exceed 1 year [41,42]. The role of gender differences also remains unresolved, as findings vary between universal associations across genders [43] and female-specific associations [44]. Furthermore, previous reviews have predominantly focused on high-income Western countries [1,30,33,35], which may under-represent settings in other countries where work-family conflict is shaped by different labour-market institutions, family policies, and cultural norms.

Accordingly, we conducted a systematic review and meta-analysis of longitudinal studies with stratification of confounding adjustment conditions to assess the association between work-family conflict and mental health and to explore potential factors influencing this association, for more reliable and generalisable conclusions. We hypothesised that both work-to-family and family-to-work conflict would be associated with poorer subsequent mental health.

## Methods

### Search strategy

This meta-analysis followed the Preferred Reporting Items for Systematic Reviews and Meta-Analyses (PRISMA) and Meta-analysis of Observational Studies in Epidemiology (MOOSE) reporting guidelines (PRISMA and MOOSE checklists presented in S1, S2, and S3 Checklists), and was registered with the International Prospective Register of Systematic Reviews (PROSPERO; CRD42022325540) [45]. The literature search adhered to the Population, Exposure, Comparator, Outcomes, and Study characteristics (PICOS) strategy, which aimed at identifying all studies examining the longitudinal associations between work-family conflict and mental health-related outcomes. The target population was set as the working adult population. The exposure was work-family conflict, which involves both work-to-family conflict and family-to-work conflict and was measured as a separate exposure. To address the inconsistent terminology in the work-family literature, we searched for any combinations between cluster (A): work-family, work-life, inter-role, multiple role; and cluster (B): conflict, balance, interfere, spillover. We also incorporated the MeSH term “Work-Life Balance” into our search strategy. Mental health outcomes included general mental well-being, general mental distress, general stress, and symptoms of depression, anxiety, post-traumatic stress disorder, acute stress disorder, suicide, non-suicidal self-harm, problem drinking, and substance use, measured by screening scales, as well as psychiatric diagnosis measured by diagnostic interviews. We searched PubMed, Web of Science, Scopus, and PsychInfo databases for papers published in English, and SinoMed for papers published in Chinese. We further performed backward citation searches by screening the reference lists of previously published systematic reviews and meta-analyses to ensure the comprehensive inclusion of all eligible studies. An initial search was conducted on May 21, 2022, with an updated search conducted on February 28, 2026, for English databases and on March 09, 2026, for the Chinese database. The full search terms based on the PICOS strategy can be found in Table A in S1 Appendix.

### Eligibility screening

Title and abstract screening was conducted on the systematic review platform Covidence [46]. Full-text screening was then conducted to confirm eligibility. The screening process was completed by two pairs of independent investigators (DL, TSM, XF, and YZ) with disagreements resolved through discussion.

Studies were deemed eligible if they met the following criteria: (1) original research applying a longitudinal design with a time lag between measurements of predictor and outcome, (2) sample from the adult working population, (3) published in English or Chinese journals, and (4) had work-to-family conflict and/or family-to-work conflict as exposure variables and mental health as an outcome variable. Studies were excluded if they only reported cross-sectional associations, only explored cross-over effects (i.e., impacts on partner), only used a combined score from summing up both directions of work-family conflict as an exposure, only reported associations between multiple waves of exposure and outcomes, such as from generalised estimating equation model, or not explicitly measuring work-family conflict raised by duties from one domain interfering with the other.

### Data extraction

Data extracted included target population, sample size, follow-up interval, measure of work-family conflict (work-to-family conflict or family-to-work conflict), mental health outcome assessment, effect sizes, standard error (SE) or 95% confidence intervals (CI), type of effect sizes, analytic method, covariates, and effect sizes by gender where available. Search results were allocated with overlapping, rotating reviewer pair assignments. For each included study, data extraction was conducted independently in parallel by two investigators (YN, TSM, XF, IHLL, MCLL, and DL). Pairings were intentionally rotated across investigators to enhance consistency in the interpretation of the extraction framework and to minimise systematic differences between pairs. Extracted entries were then cross-checked for consistency. Any discrepancies were resolved through discussion and adjudicated by a third investigator when necessary.

### Risk of bias detection

The risk of bias of each eligible paper was independently assessed by two investigators using the Newcastle-Ottawa quality assessment scale (NOS) for cohort studies [47]. The discrepancies were solved by discussion (YN, TSM, XF, IHLL, MCLL, and DL). Studies were rated based on potential bias from selection, comparability, and outcome/exposure according to the Agency for Health Research and Quality (AHRQ) standards [48]. Good quality was defined as attaining scores of 3 out of 4 for selection, 1 out of 2 for comparability, and 2 out of 3 for outcome/exposure. Fair quality was defined as a score of 2 for selection, 1 for comparability, and 2 for outcome/exposure. Poor quality was defined as scores of 0 or 1 for selection, or 0 for comparability, or 0 or 1 for outcome/exposure [48].

### Statistical analysis

All statistical analyses were conducted in R 4.5.2. Three-level random-effects meta-analyses using the generic inverse-variance weighting method were conducted with the “metafor” package to account for multiple effect sizes from the same study. In cases where only a single effect size was reported per study, two-level meta-analyses were conducted [49]. Synthesised effect sizes were quantified using standardised regression coefficients (β) and their 95% CI, in which the standardised coefficient β = 1 means a change of one standard deviation (SD) in the score of work-family conflict is associated with a change of one SD in the score of the mental health outcome. Significant effects are identified by 95% CI excluding 0, and *p*-value < 0.05. Studies using logistic regression with categorical outcomes were not involved in the meta-analysis due to the limited number of studies reporting comparable estimates. Heterogeneity across studies was indicated by *I*^*2*^ values [50]. *I*^*2*^ values below 25%, between 25%–75% and over 75% represent low, moderate, and high heterogeneity, respectively. A prediction interval was provided to facilitate interpretation of the variation between studies [51]. A meta-analysis was conducted if at least three publications with complete information regarding effect size and SE reporting the same or equivalent exposure and outcome were available. Sensitivity analyses were performed to test whether the results were consistent when each of the studies was removed (i.e., the leave-one-out approach) [52]. Further sensitivity analyses were performed only using studies with good or fair qualities defined by the AHRQ standard of NOS. All sensitivity analyses were conducted for associations with sufficient numbers of studies (*n* ≥ 3). Publication bias was assessed using the contour-enhanced funnel-plot [53], accompanied by meta-regression with SE as a covariate. B coefficients were reported from the meta-regression of publication bias, corresponding to the change in β for each SE change in work-family conflict. The “trim and fill” method was conducted at the study-level to improve the reliability of the results if significant publication bias was detected [54]. Specifically, this method was applied to an aggregated dataset with each study only having one aggregated effect size.

Effect sizes of eligible studies were converted to standardised regression coefficients (β), where possible, to ensure comparability (Table B in S1 Appendix). Effect sizes after adjusting for potential confounding factors were synthesised in a stratified approach. Given variations in the selection of confounding factors among studies, we adopted the strategy of grouping the potential confounders into different categories to facilitate comparison, including demographics, work-related factors, family-related factors, and baseline mental health (Table B in S1 Appendix). Demographics involved age, gender, socio-economic status, marital status, education level, and ethnicity. Work-related variables included working hours, job position, working overtime, job type, and other working environments. Family-related variables included the number of children, marital status, child sex, child temperament, and the number of care recipients. We then synthesised effect sizes from studies with similar adjustment conditions to induce comparability between the effect sizes being synthesised. We exclusively synthesised effect sizes with the most comprehensive confounding adjustment condition where possible, including demographics, work/family-related factors, and baseline mental health, to avoid potential inflation in the pooled results due to residual confounding from other under-adjusted effect sizes. Overall effect sizes were further reported irrespective of covariates adjusted for in the analysis.

Subgroup analyses were also conducted to assess whether the associations between work-family conflict and mental health differ across follow-up intervals (within or beyond 12 months), genders, and geographic locations. Mean difference was reported for the difference in β across groups. Subgroup analyses were only made when at least three publications provided complete information on effect size and SE, and reported the same or equivalent exposure and outcome for each subgroup.

Several refinements were made from the preregistered protocol to improve feasibility and comparability of effect estimates and to address key methodological limitations in the work-family conflict literature. First, we introduced synthesis stratified by confounding-adjustment conditions, given that the evidence base is dominated by observational studies with substantial heterogeneity in covariate sets. Second, we synthesised standardised regression coefficients (β) rather than Pearson’s r, because β corresponds to estimates from covariate-adjusted longitudinal models and therefore better supports stratification by adjustment conditions. By contrast, Pearson’s r typically reflects unadjusted or bivariate associations and is less comparable across studies with varying confounder controls. Third, we did not perform meta-analyses of odds ratios (OR) due to the limited number of studies reporting sufficiently comparable ORs. Fourth, all subgroup analyses prespecified in the protocol were conducted except for the analysis of work-family support policies, which was not feasible due to insufficient data. Finally, we added subgroup analyses by follow-up interval to address mixed evidence in studies with longer follow-up periods, as highlighted by previous reviews [41,42].

## Results

**Fig 1:**
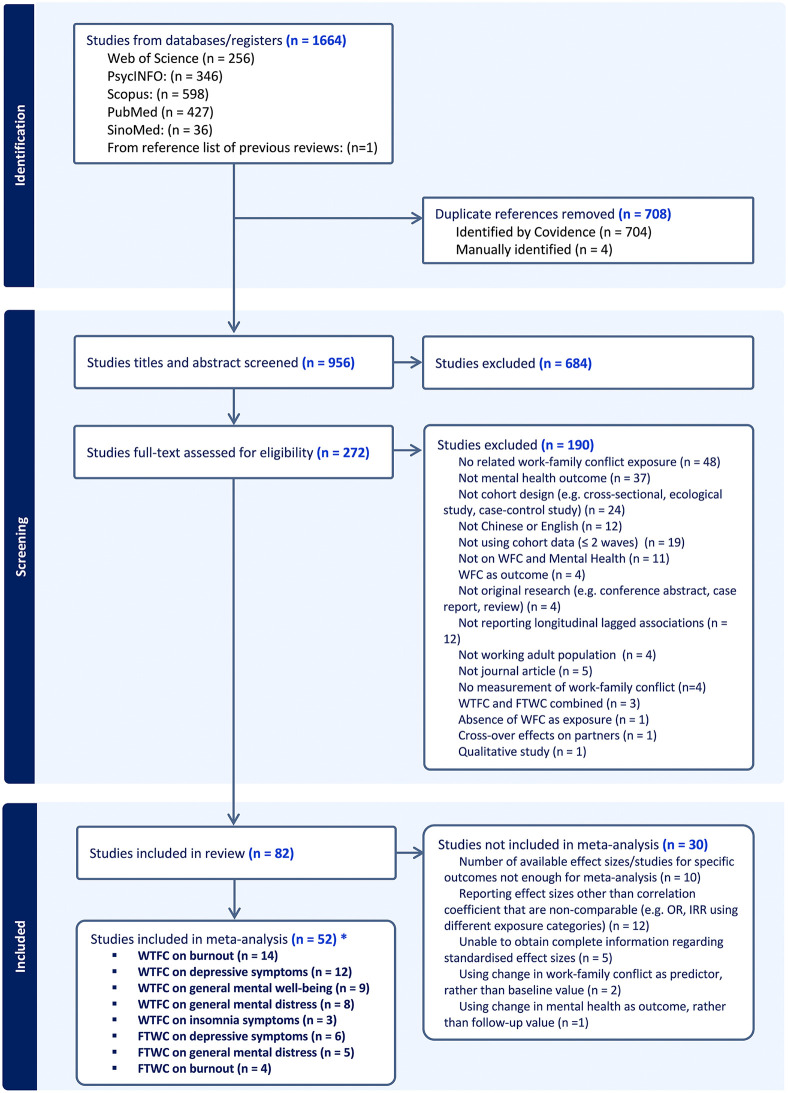
Flowchart of article screening and selection for meta-analysis. Note: *Included studies do not sum to the total as one study can contribute to multiple exposure-outcome pairs; Effect sizes for different exposure-outcome pairs in a study were analysed separately; WTFC = Work-to-Family Conflict; FTWC = Family-to-Work Conflict.

A total of 82 relevant studies reporting the longitudinal association between work-family conflict and mental health were included in the systematic review, in which 52 studies were included in the meta-analysis (Fig 1). Five mental health outcomes—depressive symptoms, burnout, general mental well-being, general mental distress, and insomnia—were available for meta-analysis. The meta-analysis included a total of 112,714 participants.

### Study characteristics

The characteristics of the 82 studies are presented in Tables C and D in S1 Appendix. Most studies were conducted in countries from North America, Europe, and Oceania (*n* = 67). Studies conducted in Asia were mostly from China (*n* = 9), followed by South Korea (*n* = 2), Japan (*n* = 2), and Israel (*n* = 1). One cross-cultural study involved participants from both China and Oceania. More studies focused on work-to-family conflict (*n* = 79) compared to family-to-work conflict (*n* = 34). The follow-up interval of the included studies ranged from 1 week to 11 years (Median = 11.0 months, IQR 11.0, 24.0 months). The relevant studies included a range of mental health outcomes, including major depressive disorder (*n* = 2), any common mental health disorders (*n* = 1), burnout (*n* = 26), depressive symptoms (*n* = 22), general mental health distress (*n* = 16) and well-being (*n* = 9), insomnia symptoms (*n* = 5), problem drinking (*n* = 5), general stress (*n* = 5), anxiety symptoms (*n* = 2), parenting stress (*n* = 2), post-traumatic stress disorder symptoms (*n* = 1), and antidepressant treatment (*n* = 1). Most studies modelled continuous outcomes of symptom severity through linear regression-based modelling (*n* = 65). Studies reporting logistic regression results (Table E in S1 Appendix) supported work-family conflict as a risk factor for subsequent general mental distress, depressive and anxiety symptoms, major depressive disorder diagnosis, and common mental disorder diagnosis [55–59].

For the 52 studies involved in the meta-analysis (Tables D and F in S1 Appendix), 40 were from North America, Europe, and Oceania. One study was cross-cultural, while studies from Asia were primarily from China (*n* = 9), with one each from Israel and South Korea. Twenty-seven of the studies had a sample size below 1,000, with 23 studies having a sample size between 1,000 and 6,000, and two studies with a sample size over 20,000. All eligible studies utilised continuous scores of work-family conflict and mental health outcomes in the analysis. Most of the studies utilised standardised scales to measure work-family conflict (*n* = 37) and mental health outcomes (*n* = 49). Regarding confounding adjustment, 14 of the studies adopted comprehensive adjustment, including sociodemographic variables, work-related resources and demands, family-related resources and demands, and baseline mental health. An additional 17 studies adjusted for baseline mental health, whereas seven did not mention any confounding adjustment. Among the studies included in the meta-analysis of depressive symptoms, 61.5% applied a comprehensive confounding adjustment set, including baseline mental health and other potential confounders. The corresponding proportions were considerably lower for burnout and general mental distress (33% each), and only two studies addressed confounding comprehensively for general mental well-being (Table F in S1 Appendix).

### Quality assessment

The quality assessment revealed that 28 (54%) of the studies included in the meta-analysis were of good or fair quality (Table G in S1 Appendix). Common features shared by poor-quality studies involve non-representative or convenience sampling, reliance on self-reported measurements, lack of baseline outcome control, and potential attrition bias from unaddressed low follow-up rates.

### Overall meta-analytic effects

#### Work-to-family conflict and mental health.

**Fig 2:**
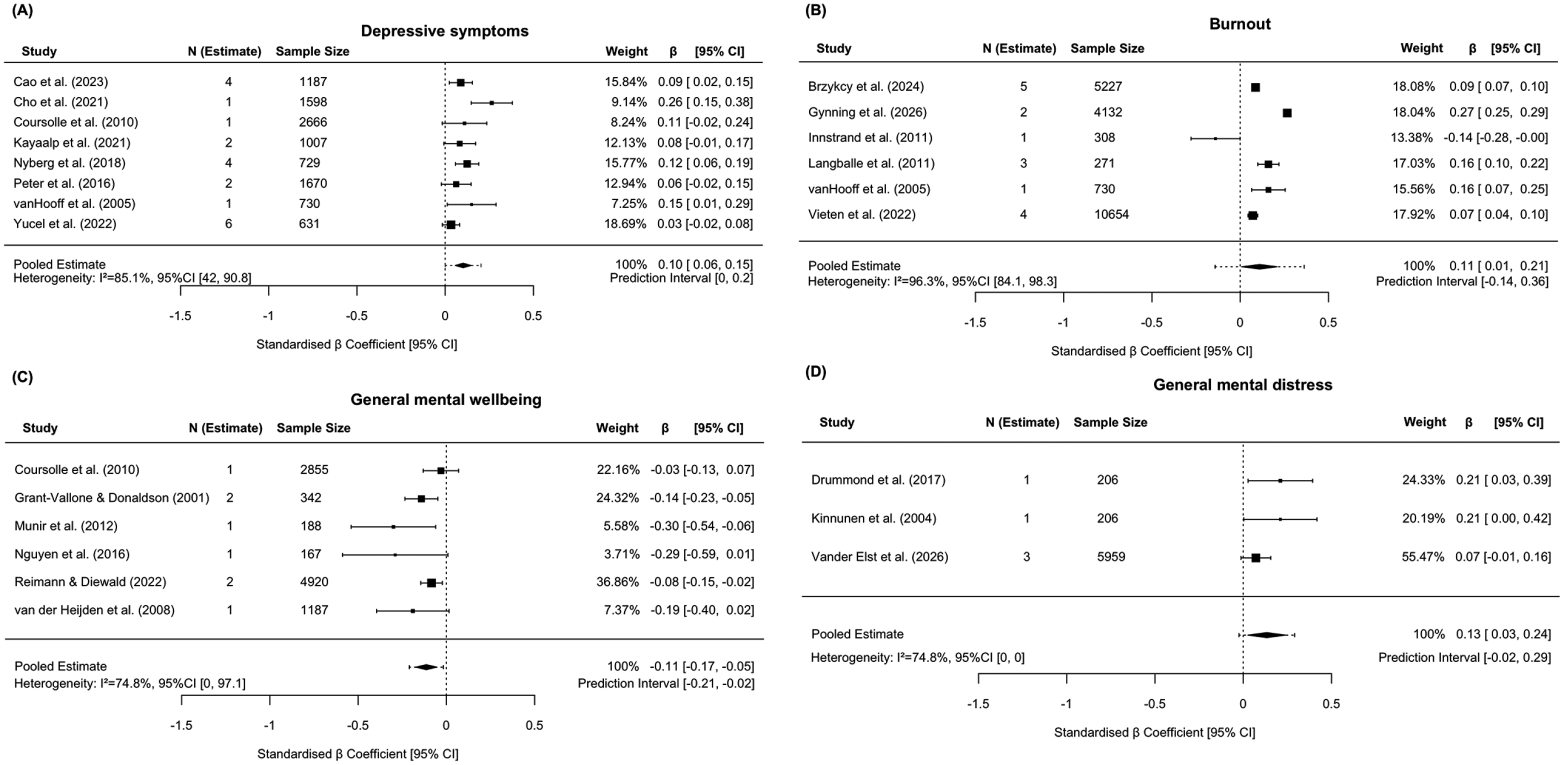
Forest plots for the association between work-to-family conflict and (A) Depressive symptoms (B) Burnout (C) General mental well-being, and (D) General mental distress, after controlling for confounders. Notes: Size of the squares indicates the weight of each individual effect size for the pooled effect. N(Estimate) = number of relevant effect sizes reported in the study. β = Standard deviation (SD) difference in the mental health outcome per 1-SD higher work–family conflict. (A) Depressive symptoms when controlling for demographics, work-related factors, and baseline mental health. (B) Burnout when controlling for demographics, work-related factors, and baseline mental health. (C) General well-being when controlling for demographics and work-related factors. (D) General mental distress when controlling for demographics, work-related factors, and baseline mental health.

The three-level meta-analysis model identified a significant overall longitudinal association between work-to-family conflict and depressive symptoms (Fig A in S1 Appendix; β = 0.11, 95% CI [0.07, 0.15]; *p* < 0.001), burnout (β = 0.20, 95% CI [0.12, 0.29]; *p* < 0.001), general mental well-being (β = −0.10, 95% CI [−0.12, −0.07]; *p* < 0.001), and general mental distress (β = 0.15, 95% CI [0.09, 0.20]; *p* < 0.001), while a non-significant association was identified for insomnia symptoms (β = 0.03, 95% CI [−0.05, 0.10]; *p* = 0.529). Large heterogeneity was identified for depressive symptoms (*I*^2^ = 89.7%, 95% CI [59.4%, 95.0%]; prediction interval [−0.03, 0.24]) but not for the other outcomes. As shown in Fig 2, the associations remained significant in studies that controlled for demographics, work-related factors, and baseline mental health for depressive symptoms (β = 0.10, 95% CI [0.06, 0.15]; *p* < 0.001), burnout (β = 0.11, 95% CI [0.01, 0.21]; *p* = 0.040), and general mental distress (β = 0.13, 95% CI [0.03, 0.24]; *p* = 0.005). The association between work-to-family conflict and depressive symptoms remained consistent in studies controlling for additional family-related factors (Fig B in S1 Appendix; β = 0.11, 95% CI [0.05, 0.16]; *p* = 0.003). In studies that controlled for available potential confounders (Fig 2), including demographics and work-related factors, the associations of work-to-family conflict with general mental well-being (β = −0.11, 95% CI [−0.17, −0.05]; *p* = 0.009) remained significant.

#### Family-to-work conflict and mental health.

**Fig 3:**
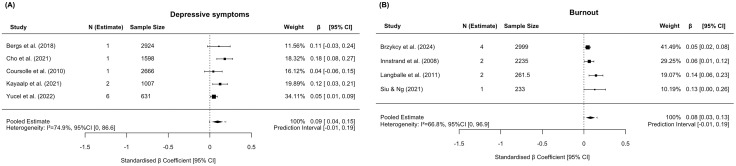
Forest plot for the association between family-to-work conflict and (A) Depressive symptoms, (B) Burnout, after controlling for confounders. Notes: Size of the squares indicates the weight of each individual effect size for the pooled effect. N(Estimate) = number of relevant effect sizes reported in the study. β  =  Standard deviation (SD) difference in the mental health outcome per 1-SD higher work–family conflict. Confounders involved demographics, work-related factors, and baseline mental health.

Family-to-work conflict was longitudinally associated with depressive symptoms (Fig C in S1 Appendix; β = 0.10, 95% CI [0.05, 0.16]; *p* = 0.003) and general mental distress (β = 0.12, 95% CI [0.05, 0.20]; *p* = 0.021), but not with burnout (β = 0.06, 95% CI [−0.00, 0.12]; *p* = 0.086). Large heterogeneity was identified for depressive symptoms (*I*^2^ = 74.0%, 95% CI [17.2%, 86.3%]; prediction interval [0.00, 0.20]), burnout (*I*^2^ = 81.9%, 95% CI [0%, 96.9%]; prediction interval [−0.08, 0.20]), and general mental distress (*I*^2^ = 84.2%, 95% CI [51.5%, 93.5%]; prediction interval [−0.05, 0.29]). Among studies controlling for demographics, work-related factors, and baseline mental health (Fig 3), the associations are significant for both depressive symptoms (β = 0.09, 95% CI [0.04, 0.15]; *p* = 0.006) and burnout (β = 0.08, 95% CI [0.03, 0.13]; *p* = 0.013).

#### Sensitivity analyses.

The sensitivity analyses revealed a generally stable relationship between work-family conflict and mental health. The leave-one-out meta-analysis (Table H in S1 Appendix) showed that most associations remained consistent with the overall results, except for the association between work-to-family conflict and general mental distress, which moderately attenuated when removing one study (Kinnunen and colleagues, 2004) while remaining significant [38]. The association between family-to-work conflict and burnout turned significant when removing one study (Ren and colleagues, 2025) [60], while the association between family-to-work conflict and general mental distress turned nonsignificant when removing one study (Matthews and colleagues, 2014) [61]. Sensitivity analyses after removing studies of poor-quality also showed that the associations between work-family conflict and mental health outcomes remained generally consistent (Table I in S1 Appendix), except that the association between family-to-work conflict and general mental distress became nonsignificant (β = 0.13, 95% CI [−0.02, 0.28]; *p* = 0.077), while the pooled effect size for work-to-family conflict and burnout attenuated but remained significant (β = 0.12, 95% CI [0.06, 0.18]; *p* = 0.001).

Regarding publication bias, funnel-plots showed asymmetrical distribution of effects for most of the work-to-family conflict and mental health pairs, except for the associations with insomnia (Fig D in S1 Appendix). The distributions of family-to-work conflict-related effects are approximately symmetrical (Fig E in S1 Appendix) except for the association with general mental distress. The meta-regression models with SE as covariate identified significant publication bias for the association of work-to-family conflict with depressive symptoms (B = 1.46, SE = 0.63, *p* = 0.028), burnout (B = 2.05, SE = 0.58, *p* = 0.001), general mental well-being (B = −1.41, SE = 0.60, *p* = 0.039), and general mental distress (B = 3.11, SE = 0.55, *p* < 0.001), as well as the association of family-to-work conflict with general mental distress (B = 3.34, SE = 0.11, *p* = 0.014). The trim-and-fill analysis was applied, which balanced the funnel plot at the study-level (Figs F and G in S1 Appendix) and yielded similar results for the association of work-to-family conflict with depressive symptoms (β = 0.10, 95% CI [0.06, 0.14]; *p* < 0.001), burnout (β = 0.19, 95% CI [0.14, 0.23]; *p* < 0.001), and general mental well-being (β = −0.09, 95% CI [−0.12, −0.07]; *p* < 0.001), and general mental distress (β = 0.13, 95% CI [0.09, 0.16]; *p* = 0.008). The association between family-to-work conflict and general mental distress attenuated moderately but remained significant (β = 0.09, 95% CI [0.00, 0.18]; *p* = 0.049).

### Subgroup comparison

*By follow-up interval:* Subgroup comparisons between follow-up intervals of over and within 1 year were available for depressive symptoms and burnout. As shown in Fig H in S1 Appendix, meta-regression results indicated no significant difference in the association between work-to-family conflict and depressive symptoms (mean difference = −0.02, 95% CI [−0.08, 0.04]; *p* = 0.468) across studies with over (β = 0.12, 95% CI [0.06, 0.17]; *p* < 0.001) or within (β = 0.08, 95% CI [0.04, 0.13]; *p* < 0.001) 1 year of follow-up interval. No significant difference was found in the association between work-to-family conflict and burnout (mean difference = 0.17, 95% CI [−0.02, 0.36]; *p* = 0.080) across studies with over (β = 0.07, 95% CI [−0.06, 0.20]; *p* = 0.269) and within (β = 0.24, 95% CI [0.15, 0.34]; *p* < 0.001) 1 year of follow-up interval. When comparing follow-up intervals of over and within 2 and 3 years, no significant difference emerged in the association between work-to-family conflict and depressive symptoms or general mental well-being (Fig I in S1 Appendix).

Apart from the above comparison, different combinations of exposures and outcomes are presented for each follow-up interval due to study availability. In studies with a follow-up interval of over 1 year (Fig J in S1 Appendix), our results indicated significant associations between work-to-family conflict and general mental well-being (β = −0.08, 95% CI [−0.12, −0.05]; *p* = 0.004). A significant association was also found for family-to-work conflict and depressive symptoms (β = 0.10, 95% CI [0.03, 0.16]; *p* = 0.012). When the follow-up interval was between six and 12 months (Fig K in S1 Appendix), there were significant associations of work-to-family conflict and depressive symptoms (β = 0.08, 95% CI [0.04, 0.13]; *p* < 0.001) and burnout (β = 0.19, 95% CI [0.11, 0.27]; *p* < 0.001). With a follow-up interval of less than six months (Fig K in S1 Appendix), work-to-family conflict (β = 0.10, 95% CI [0.06, 0.15]; *p* = 0.003) and family-to-work conflict (β = 0.14, 95% CI [0.04, 0.24]; *p* = 0.033) were significantly associated with general mental distress.

**Fig 4:**
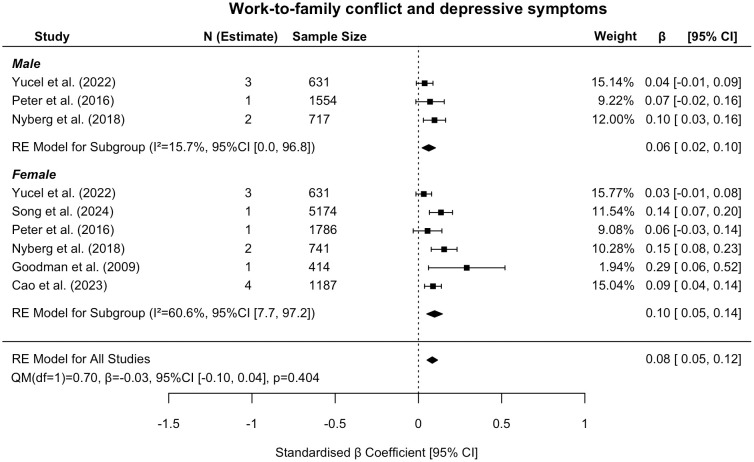
Forest plots for the association between work-to-family conflict and depressive symptoms by gender. Note: N(Estimate) = number of relevant effect sizes reported in the study. β  =  Standard deviation (SD) difference in the mental health outcome per 1-SD higher work–family conflict. β for subgroup comparison indicates the mean difference in pooled effects between sex subgroups, with females as the reference group. Pooled effects from studies in all control conditions.

*By gender:* Gender-specific associations were only available for depressive symptoms (Fig 4). The results from meta-regression indicated that there was no significant between-gender difference (mean difference = −0.03, 95% CI [−0.10, 0.04]; *p* = 0.404) in the association between work-to-family conflict and depressive symptoms in males (β = 0.06, 95% CI [0.02, 0.10]; *p* < 0.001) and females (β = 0.10, 95% CI [0.05, 0.14]; *p* < 0.001).

**Fig 5:**
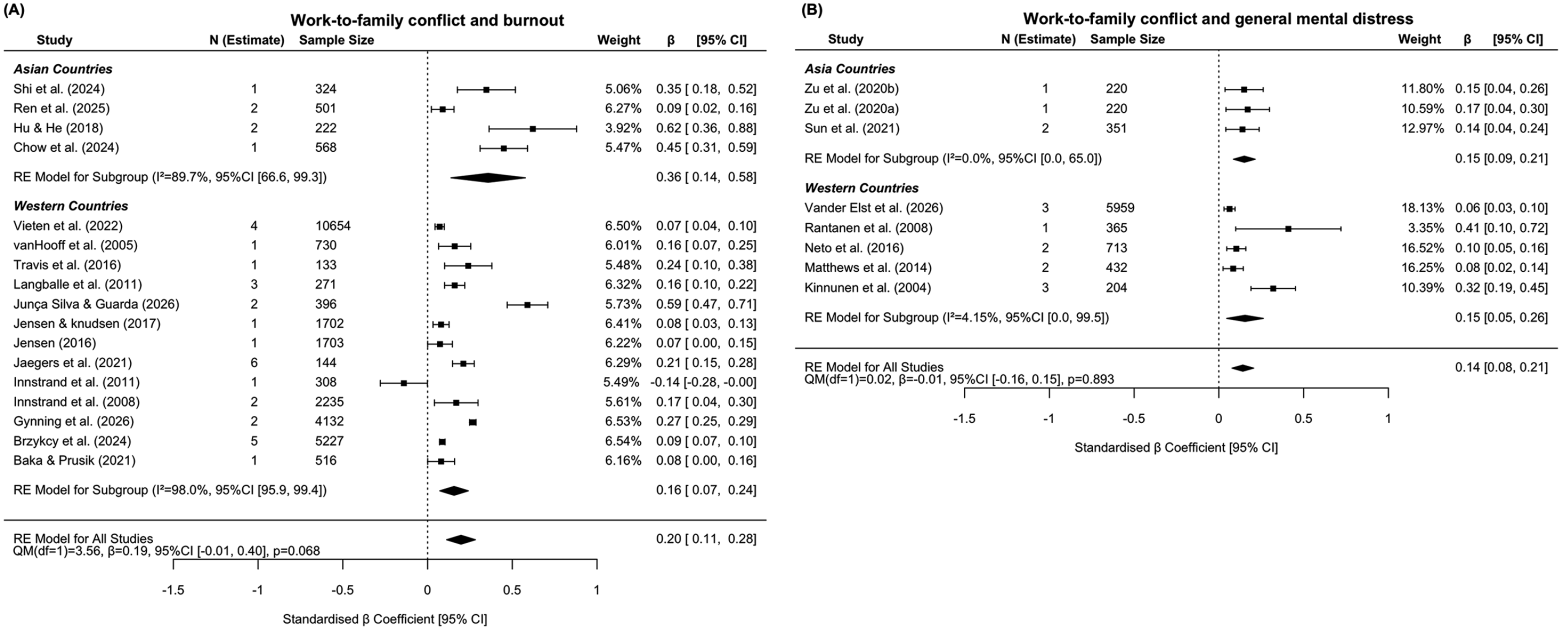
Forest plots for the association between work-to-family conflict and mental health by geographical location. (A) Burnout, (B) General mental distress. Note: N(Estimate) = number of relevant effect sizes reported in the study. β = Standard deviation (SD) difference in the mental health outcome per 1-SD higher work–family conflict. β for subgroup comparison indicates the mean difference in pooled effects between geographical location subgroups, with Western countries as the reference group. Available Asian studies were all from Chinese populations. Available Western studies were from Germany, the Netherlands, Norway, Poland, Portugal, Sweden, and the US for burnout, and from Belgium, Finland, Portugal, the US, and Finland for general mental distress. Pooled effects from studies in all control conditions.

*By geographical location:* The comparison by geographical location was available for burnout and general mental distress (Fig 5A, 5B). Available Asian studies were all from Chinese populations. Available Western studies consisted of participants from Norway, Sweden, the Netherlands, Germany, Finland, Portugal, and the US. Meta-regression indicated a stronger association between work-to-family conflict and burnout in Asian (β = 0.36, 95% CI [0.14, 0.56]; *p* = 0.001) than in Western countries (β = 0.16, 95% CI [0.07, 0.24]; *p* < 0.001), but no significant difference was detected between Asian and Western settings (mean difference = 0.19, 95% CI [−0.01, 0.40]; *p* = 0.068). No cross-geographical area difference appeared (mean difference = −0.01, 95% CI [−0.16, 0.15] *p* = 0.893) in the association between work-to-family conflict and general mental distress between Asian (β = 0.15, 95% CI [0.09, 0.21]; *p* < 0.001) and Western countries (β = 0.15, 95% CI [0.05, 0.26]; *p* = 0.005). We performed a sensitivity analysis excluding US studies to assess cross-geographical differences. This sensitivity analysis did not change our overall results (Fig L in S1 Appendix).

## Discussion

This systematic review and meta-analysis synthesised longitudinal evidence on the association between work-family conflict and mental health, accounting for confounding adjustment. Work-to-family conflict was associated with depressive symptoms, burnout, and general mental distress, while family-to-work conflict was associated with depressive symptoms and burnout. These associations persisted after adjustment for demographics, work-related factors, and baseline mental health. Our findings align with the Work-Home Resources model [20], which frames work-family conflict as a resource-loss process in which demands in one domain deplete shared personal resources, contributing to stress and poor mental health.

Our study identified small effect sizes (standardised β from 0.08 to 0.13) compared with those reported in a previous meta-analysis of mostly cross-sectional and unadjusted associations (Pearson’s r from 0.22 to 0.38) [21]. Effect sizes were further attenuated in studies with more comprehensive confounding adjustment, particularly for work-to-family conflict and burnout (standardised β attenuated from 0.20 to 0.11), likely reflecting shared sociodemographic, work, and family-related confounders [23,26–29]. Moreover, longitudinal designs may lead to smaller effect sizes since the impact of work stressors on well-being frequently peak after two to three years before declining [62]. Excluding lower-quality studies also reduced effect sizes, possibly due to weaker confounding control or selection bias from nonrepresentative samples. Nevertheless, our pooled effect sizes are comparable to those of other well-established mental health risk factors (e.g., stressful life events and socio-economic status) [63,64], and would have a major public health impact given the large populations worldwide affected by work-family conflict [65,66].

Our systematic review highlighted several important gaps in our understanding of work-family conflict and mental health. First, most studies focused on depressive symptoms, burnout, and general stress, with limited evidence on other outcomes (e.g., anxiety, PTSD, and problem drinking). Most studies examined symptom severity rather than incident mental health conditions. Only one study explored common mental disorders [59], and two examined the onset or recurrence of major depressive disorders [57,58]. Second, baseline mental health–adjusted synthesis was possible only for work-to-family conflict with depressive symptoms, burnout, and general mental distress and for family-to-work conflict with depressive symptoms and burnout. Even longitudinal designs cannot entirely rule out the possibility of reverse causality. Adjusting for baseline mental health in longitudinal research helps alleviate this concern and provides some of the strongest available evidence for causal inference [67]. Nevertheless, only a few studies applied a comprehensive adjustment for baseline mental health and other confounders, particularly for outcomes beyond depression, highlighting the need for higher-quality longitudinal research in this field. Third, family-to-work conflict remains under-explored, as evidence for general mental distress and well-being is limited and often lacks adequate adjustment. Fourth, publication bias was evident for multiple outcomes. While bias-adjusted estimates generally supported the main findings, the association between family-to-work conflict and burnout was attenuated and should be interpreted cautiously. Furthermore, future research should also move beyond treating social determinants, such as gender, SES, marital status, and parental status, only as covariates, and examine how these factors shape both exposure and vulnerability to work-family conflict. This is particularly important given inequalities in paid and unpaid work [68–71]. Finally, more studies are needed across different time periods, regions, and policy contexts to assess how changing work conditions, gender norms, demographic patterns, and labour and family policies influence work-family dynamics [72–77].

Our meta-analysis also explored the potential role of follow-up interval, gender, and geographical location. Associations were generally consistent across follow-up intervals, suggesting long-lasting mental health implications [2]. Work-to-family conflict was also associated with depressive symptoms in both women and men, indicating that its mental health impact is not limited to one gender. This may reflect women’s continuing disproportionate caregiving responsibilities and men’s increasing involvement in care despite workplace policies that often remain shaped by traditional gender norms [6,78–80]. Meta-regression identified no significant regional differences in the associations of work-to-family conflict with burnout or general mental distress, suggesting broadly consistent adverse mental health consequences across settings. Nevertheless, differences in family policies, workplace norms, and caregiving expectations may still shape exposure and vulnerability, warranting further cross-national research [81–83]. However, it should be noted that the Asian subgroup included only Chinese populations due to the scarcity of studies in other countries, highlighting the need for studies from broader cultural and socio-political contexts to improve generalisability.

Our study is subject to several limitations. First, substantial heterogeneity was observed across studies [84]. However, sensitivity analyses, including individual-study influence checks, exclusion of poor-quality studies, and trim-and-fill analysis, produced consistent results. Gender subgroup analyses showed that heterogeneity remained significant among females but not males, indicating a need for more high-quality studies with comparable measures, designs, and outcomes to examine sources of heterogeneity among females. Second, subgroup analyses may be unstable because of the limited number of studies, although stratified results were provided to support interpretation. Publication bias is also a concern, as funnel-plot asymmetry and small-study effects were detected for several associations. Although trim-and-fill analyses produced broadly similar estimates, bias cannot be ruled out, especially where few studies were available. Moreover, pooled standardised beta coefficients are influenced by study-specific covariate adjustment. Nevertheless, pooling estimates with broadly comparable demographic, work, family, and baseline mental health adjustments improved consistency and reduced bias compared with bivariate correlations. Given that existing studies rely on observational data, synthesising effect sizes with consideration of confounding adjustment conditions provides more reliable results compared to bivariate correlations, reducing the risk of bias and enhancing the validity of our findings. Future quasi-experimental studies with matched exposed and unexposed groups could strengthen causal evidence [85,86].

By synthesising longitudinal studies adjusting for baseline outcomes and confounders, this review provides a rigorous synthesis of evidence demonstrating that work-to-family conflict and family-to-work conflict are associated with mental health outcomes, highlighting work-family conflict as an important social determinant of mental health. Our results have important implications for policy and practice. Reducing work-family conflict requires both population-level and workplace-level strategies. Policy responses should include promoting gender-equitable caregiving norms, encouraging men’s use of parental leave, reforming parental leave policies, expanding provision of care to children and older adults, supporting flexible work, protecting work-home boundaries through measures such as “right to disconnect” laws, and considering shorter working hours where feasible [7,87–92]. At the organisational level, family-supportive supervisor behaviours, remote or hybrid work options, predictable and flexible scheduling, childcare support, and integration of work-family conflict into mental health risk assessments and workplace interventions may help employees balance work and caregiving responsibilities [8,93–96]. These approaches should be designed carefully to support both worker well-being and sustainable productivity and tailored to socio-political contexts, including cultural and gender norms, parental leave systems, long-term care provision, and organisational support [6,7,79,80,97]. Collective efforts leveraging the countervailing trends, including the rise of telecommuting, shifting gender norms, and signs of increased worker unionisation, could help working populations to reconcile work and family spheres, ultimately enhancing their overall mental well-being [75,98,99].

### Disclosure of generative AI usage

During the preparation of this work, the authors used ChatGPT-5.4 to improve language. After using this tool, the authors reviewed and edited the content as needed and take full responsibility for the content of the publication.

## Supporting information

S1 Appendix Supplementary Tables and Figures. (DOCX)

S1 Checklist PRISMA 2020 checklist. PRISMA 2020 Checklist. Page MJ, McKenzie JE, Bossuyt PM, Boutron I, Hoffmann TC, Mulrow CD, and colleagues. The PRISMA 2020 statement: an updated guideline for reporting systematic reviews. BMJ 2021;372:n71. https://doi.org/10.1136/bmj.n71. This work is licensed under CC BY 4.0. To view a copy of this license, visit [https://creativecommons.org/licenses/by/4.0/].
(DOCX)

S2 Checklist PRISMA 2020 for abstracts checklist. PRISMA 2020 for Abstracts Checklist. Page MJ, McKenzie JE, Bossuyt PM, Boutron I, Hoffmann TC, Mulrow CD, and colleagues. The PRISMA 2020 statement: an updated guideline for reporting systematic reviews. BMJ 2021;372:n71. https://doi.org/10.1136/bmj.n71. This work is licensed under CC BY 4.0. To view a copy of this license, visit [https://creativecommons.org/licenses/by/4.0/].
(DOCX)

S3 Checklist Meta-analysis of observational studies in epidemiology checklist. (DOCX)

## Abbreviations

AHRQ: Agency for Health Research and Quality
CI: confidence intervals
NOS: Newcastle-Ottawa Scale
OR: odds ratios
SD: standard deviation
SE: standard error.
