# Supplementary material for: Work-family conflict and mental health: A systematic review and meta-analysis
Source: PLoS Med. 2026 Jul 17;23(7):e1005162. doi: 10.1371/journal.pmed.1005162 (PMC13379121; doi:10.1371/journal.pmed.1005162)
Supplement: S1 Appendix — (DOCX) [file pmed.1005162.s001.docx]

**S1 Appendix**

Table of Contents

[Table A. Search terms developed based on the PECOS strategy 3](#_Toc233887136)

[Table B. Detailed procedure of data cleaning and statistics 5](#_Toc233887137)

[Table C. Summaries of published longitudinal studies of work-family conflict and mental health 6](#_Toc233887138)

[Table D. Characteristics of published studies assessing longitudinal association between work-family conflict and mental health outcomes 7](#_Toc233887139)

[Table E. Characteristics of studies reporting logistic regression of longitudinal association between work-family conflict and mental health outcomes 13](#_Toc233887140)

[Table F. Confounding adjustment conditions of the articles included in meta-analysis 14](#_Toc233887141)

[Table G. Quality check for the articles included in meta-analysis 17](#_Toc233887142)

[Table H. Sensitivity test of meta-analysis results through removing one study each time 22](#_Toc233887143)

[Table I. Sensitivity test without studies with poor quality 24](#_Toc233887144)

[*Fig A*. Forest plots for the association between work-to-family conflict and (A) Depressive symptoms, (B) Burnout, (C) General mental wellbeing, (D) General mental distress, (E) Insomnia symptoms. 25](#_Toc233887145)

[*Fig B.* Forest plot for the relationship between work-to-family conflict and depressive symptoms after adjusting for demographics, work-related factors, family-related factors, and baseline mental health 26](#_Toc233887146)

[*Fig C.* Forest plots for the association between family-to-work conflict and (A) Depressive symptoms, (B) Burnout, (C) General mental distress. 27](#_Toc233887147)

[*Fig D.* Contour-enhanced funnel plot of publication bias for the relationship between work-to-family conflict and (A) Depressive symptoms, (B) Burnout, (C) General mental wellbeing, (D) General mental distress, (E) Insomnia symptoms. 28](#_Toc233887148)

[*Fig E.* Contour-enhanced funnel plot of publication bias for the relationship between family-to-work conflict and (A) Depressive symptoms, (B) Burnout, (C) General mental distress. 29](#_Toc233887149)

[*Fig F.* Contour-enhanced funnel plot of publication bias for the relationship between work-to-family conflict and (A) Depressive symptoms, (B) Burnout, (C) General mental well-being, (D) General mental distress, after applying the trim-and-fill method at study-level. 30](#_Toc233887150)

[*Fig G.* Contour-enhanced funnel plot of publication bias for the relationship between family-to-work conflict and general mental distress, after applying the trim-and-fill method at study-level. 31](#_Toc233887151)

[*Fig H.* Work-to-family conflict and mental health by follow-up length within or over one year. (A) Work-to-family conflict and depressive symptoms, (B) Work-to-family conflict and burnout 32](#_Toc233887152)

[*Fig I.* Work-to-family conflict and mental health by follow-up length with cut-offs of two and three years. (A) Work-to-family conflict and depressive symptoms with a three-year cut-off, (B) Work-to-family conflict and general mental wellbeing with a two-year cut-off. 33](#_Toc233887153)

[*Fig J.* Forest plot for the relationship between work-family conflict and mental health with follow-up interval over 12 months. (A) Work-to-family conflict and general mental wellbeing. (B) Family-to-work conflict and depressive symptoms. 34](#_Toc233887154)

[*Fig K.* Forest plot for the relationship between work-family conflict and mental health with follow-up interval within 12 months. (A) Work-to-family conflict and general mental distress (B) Family-to-work conflict and general mental distress. (C) Work-to-family conflict and depressive symptoms. (D) Work-to-family conflict and burnout. 35](#_Toc233887155)

[*Fig L.* Forest plots for the association between work-to-family conflict and mental health by geographical location after excluding US studies. (A) Burnout, (B) General mental distress. 37](#_Toc233887156)

| Table A. Search terms developed based on the PECOS strategy | |
| --- | --- |
| Database | **Search terms** |
| PubMed | ("Work-Life Balance"[Mesh] OR "work-family conflict*"[Title/Abstract] OR "work family conflict*"[Title/Abstract] OR "work-to-family conflict*"[Title/Abstract] OR "work-to-family interfer*"[Title/Abstract] OR "work-family interfer*"[Title/Abstract] OR "family-to-work conflict*"[Title/Abstract] OR "family-to-work interfer*"[Title/Abstract] OR "work-family spillover*"[Title/Abstract] OR "interrole conflict*"[Title/Abstract] OR "multiple role stress*"[Title/Abstract] OR "work-life balance*"[Title/Abstract] OR "work-family balance*"[Title/Abstract] OR "work-life conflict*"[Title/Abstract] OR "work-life interfer*"[Title/Abstract])  AND  ("Mental Health"[Mesh] OR "Mental Disorders"[Mesh] OR "Stress, Psychological"[Mesh] OR "Depression"[Mesh] OR "Depressive Disorder"[Mesh] OR "Anxiety"[Mesh] OR "Anxiety Disorders"[Mesh] OR "Stress Disorders, Post-Traumatic"[Mesh] OR "Stress Disorders, Traumatic, Acute"[Mesh] OR "Suicide"[Mesh] OR "Self-Injurious Behavior"[Mesh] OR "Substance-Related Disorders"[Mesh] OR "mental health"[Title/Abstract] OR "mental well-being"[Title/Abstract] OR "mental wellbeing"[Title/Abstract] OR "mental illness*"[Title/Abstract] OR "mental disorder*"[Title/Abstract] OR "psychologic*"[Title/Abstract] OR "distress*"[Title/Abstract] OR "depress*"[Title/Abstract] OR "anxiet*"[Title/Abstract] OR "PTSD"[Title/Abstract] OR "PTSS"[Title/Abstract] OR "posttraumatic stress*"[Title/Abstract] OR "post-traumatic stress*"[Title/Abstract] OR "acute stress*"[Title/Abstract] OR "suicid*"[Title/Abstract] OR "self-harm*"[Title/Abstract] OR "psychiatric*"[Title/Abstract] OR "alcohol*"[Title/Abstract] OR "substance*"[Title/Abstract])  AND ("Longitudinal Studies"[Mesh] OR "Cohort Studies"[Mesh] OR "Prospective Studies"[Mesh] OR "Follow-Up Studies"[Mesh] OR "longitudin*"[Title/Abstract] OR "cohort*"[Title/Abstract] OR "panel*"[Title/Abstract] OR "cross-lagged"[Title/Abstract] OR "prospective*"[Title/Abstract] OR "follow-up"[Title/Abstract]) |
| Web of Sciences | (AB=("Work-family conflict*") OR AB=("work and family conflict*") OR AB=("work-to-family conflict*") OR AB=("work/family conflict*") OR AB=("work-to-family interference*") OR AB=("work-family interference*") OR AB=("family-to-work conflict*") OR AB=("family-to-work interference*") OR AB=("work-family spillover*") OR AB=("interrole conflict*") OR AB=("multiple role stress*") OR AB=("work-life balance*") OR AB=("work-family balance*"))  AND  (AB=("Mental health*") OR AB=("mental well-being*") OR AB=("mental wellbeing*") OR AB=("mental illness*") OR AB=("mental disorder*") OR AB=(psychological*) OR AB=("psychological well-being*") OR AB=("psychological wellbeing*") OR AB=("psychological symptoms*") OR AB=("psychological strain*") OR AB=(distress*) OR AB=(depression*) OR AB=(depressive*) OR AB=(anxiety*) OR AB=(PTSD*) OR AB=(PTSS*) OR AB=("posttraumatic stress symptoms*") OR AB=("acute stress disorder*") OR AB=("acute stress symptoms*") OR AB=(suicide*) OR AB=(suicidality*) OR AB=( suicidal*) OR AB=("deliberate self-harm*") OR AB=("psychiatric diagnoses*") OR AB=(alcohol*) OR AB=("substance use*"))  AND  (AB=(Longitudin*) OR AB=(cohort*) OR AB=(panel*) OR AB=(cross-lagged*) OR AB=(follow-up)) |
| Scopus | ( TITLE-ABS-KEY ( "work-family conflict*" OR "work and family conflict*" OR "work-to-family conflict*" OR "work/family conflict*" OR "work-to-family interfer*" OR "work-family interfer*" OR "family-to-work conflict*" OR "family-to-work interfer*" OR "work-family spillover*" OR "interrole conflict*" OR "multiple role stress*" OR "work-life balance*" OR "work-family balance*" ))  AND  ( TITLE-ABS-KEY ( "mental health" OR "mental well-being" OR "mental wellbeing" OR "mental illness*" OR "mental disease*" OR "mental disorder*" OR "psychiatric disorder*" OR psychologic* OR "psychologic* well-being" OR "psychologic* wellbeing" OR "psychologic* symptom*" OR "psychologic* strain*" OR distress* OR depress* OR anxiet* OR PTSD OR PTSS OR "posttraumatic stress*" OR "post-traumatic stress*" OR "acute stress*" OR suicid* OR "deliberate self-harm*" OR "psychiatric diagnos*" OR alcohol* OR "substance use*" OR "substance abuse*" ))  AND  ( TITLE-ABS-KEY ( longitudin* OR cohort* OR panel* OR "cross-lagged" OR "follow-up" )) |
| PsychInfo | (tiab("work-family conflict*") OR tiab("work and family conflict*") OR tiab("work-to-family conflict*") OR tiab("work/family conflict*") OR tiab("work-to-family interfer*") OR tiab("work-family interfer*") OR tiab("family-to-work conflict*") OR tiab("family-to-work interfer*") OR tiab("work-family spillover*") OR tiab("interrole conflict*") OR tiab("multiple role stress*") OR tiab("work-life balance*") OR tiab("work-family balance*") OR SU("Family Work Relationship") OR SU("Work Life Balance") OR SU("Role Conflicts"))  AND  (tiab("mental health") OR tiab("mental well-being") OR tiab("mental wellbeing") OR tiab("mental illness*") OR tiab("mental disorder*") OR tiab(psychologic*) OR tiab("psychologic* well-being") OR tiab("psychologic* wellbeing") OR tiab("psychologic* symptom*") OR tiab("psychologic* strain*") OR tiab(distress*) OR tiab(depress*) OR tiab(anxiet*) OR tiab(PTSD) OR tiab(PTSS) OR tiab("posttraumatic stress*") OR tiab("post-traumatic stress*") OR tiab("acute stress*") OR tiab(suicid*) OR tiab("deliberate self-harm*") OR tiab("psychiatric diagnos*") OR tiab(alcohol*) OR tiab("substance use*") OR SU("Mental Health") OR SU("Mental Disorders") OR SU("Well Being") OR SU("Psychological Stress") OR SU("Major Depression") OR SU("Anxiety") OR SU("Posttraumatic Stress Disorder") OR SU("Suicide") OR SU("Substance Use Disorder"))  AND  (tiab(longitudin*) OR tiab(cohort*) OR tiab(panel*) OR tiab("cross-lagged") OR SU("Longitudinal Studies") OR SU("Followup Studies")) |
| SinoMed  (in Chinese) | (( "家庭-工作"[常用字段:智能] OR "工作-家庭"[常用字段:智能] OR "家庭工作"[常用字段:智能] OR "工作家庭"[常用字段:智能] OR "工作生活"[常用字段:智能] OR "生活工作"[常用字段:智能] OR "工作-生活"[常用字段:智能] OR "生活-工作"[常用字段:智能] OR "多重角色"[常用字段:智能] OR "多角色"[常用字段:智能] OR "角色冲突"[常用字段:智能] OR "职业紧张"[常用字段:智能]))  AND  ("心理"[常用字段:智能] OR "精神"[常用字段:智能] OR "抑郁"[常用字段:智能] OR "焦虑"[常用字段:智能] OR "紧张"[常用字段:智能] OR "应激障碍"[常用字段:智能] OR "压力"[常用字段:智能] OR "痛苦"[常用字段:智能] OR "自杀"[常用字段:智能] OR "自残"[常用字段:智能] OR "物质滥用"[常用字段:智能] OR "酒精"[常用字段:智能])  AND  ("纵向"[常用字段:智能] OR "队列"[常用字段:智能] OR "面板"[常用字段:智能] OR "交叉滞后"[常用字段:智能]) |

PECOS: Population, Exposure, Comparator, Outcomes, and Study characteristics

| **Table B. Detailed procedure of data cleaning and statistics** | |
| --- | --- |
| **Transformation of effect size** | For studies reporting unstandardised coefficients and SE, the standardised coefficient and SE were calculated through the unstandardised coefficient, SE, and standard deviations of the exposure and outcome variables.^1^ For studies not reporting the SE, the SE was calculated through the standardised coefficient, sample size, number of covariates, and *p*-value.^2^ |
| **Pooled effect of studies adjusting for confounding factors** | Potential confounding variables were categorised into four domains, including demographics, work-related factors, family-related factors, and baseline mental health. Studies/effect sizes with the most comprehensive confounding adjustment conditions (i.e. adjusted for confounders from all four domains) were synthesised together. In cases where there were not enough studies adjusting for all aspects of confounding factors, studies that adjusted for demographics and work-related factors were analysed. |
| **Three-level generic inverse-variance weighting meta-analyses model (R code)** | library(metafor)  Meta_model <- rma.mv(yi = Beta_effect_size,  V = Variance,  slab = Study_ID,  data = Data_Set_Name,  random = ~ 1 \| Study_ID/Coefficient_ID,  test = "t",  method = "REML") |

| **Table C. Summaries of published longitudinal studies of work-family conflict and mental health** | |
| --- | --- |
|  | **Number of studies (%)**  **(Total n = 82)** |
| **Work-family conflict type** |  |
| Solely work-to-family conflict | 48 (59.2) |
| Solely family-to-work conflict | 3 (3.9) |
| Both | 31 (36.8) |
| **Mental health outcomes** ^a^ |  |
| Burnout | 26 (28.9) |
| Depressive symptoms | 22 (26.3) |
| General mental distress | 16 (19.7) |
| General mental well-being | 9 (11.8) |
| Problem drinking | 5 (5.3) |
| General stress | 5 (5.3) |
| Insomnia | 5 (5.3) |
| Anxiety symptoms | 2 (2.6) |
| Parenting stress | 2 (2.6) |
| Major Depressive Disorder | 2 (2.6) |
| Post-traumatic stress disorder (PTSD) symptoms | 1 (1.3) |
| Combined Depression & Anxiety | 1 (1.3) |
| Common mental disorders diagnosis | 1 (1.3) |
| Antidepressant treatment | 1 (1.3) |
| **Geographic location** |  |
| North America | 19 (22.4) |
| Europe | 41 (48.7) |
| Oceania | 7 (9.2) |
| Asia | 14 (19.7) |
| Cross-continents | 1 (1.3) |

^a^ The number of studies may exceed 82 as some studies examined more than one outcome.

| Table D. Characteristics of published studies assessing longitudinal association between work-family conflict and mental health outcomes | | | | | | | | | | | | | | | | | |
| --- | --- | --- | --- | --- | --- | --- | --- | --- | --- | --- | --- | --- | --- | --- | --- | --- | --- |
| Study | Work-Family Conflict Exposure | | Work-Family Conflict Measures | | Mental Health Measures | | Sample Size | | Main Analytic Methods | | Follow-up  Interval (months)^†^ | | Female  (%) | | Location | | Meta-Analysis |
| Burnout |  | |  | |  | |  | |  | |  | |  | |  | |  |
| Baka & prusik (2021)^3^ | WTFC | | Netemeyer’s (1996) scale of WFC* | | OLBI* | | 516 | | Cross-lagged panel model | | 12 | | 84 | | Poland | | + |
| Chow et al., (2024)^4^ | WTFC | | WFBS* | | BMS* | | 568 | | Mediation analysis | | 9 | | 57 | | China Mainland | | + |
| Costa et al. (2023)^5^ | WTFC | | Unstandardised continuous measure | | SMBM | | 1843 | | Logistic regression model | | 12 | | 83 | | Portugal | |  |
| Gynning et al. (2024)^6^ | WTFC | | Fisher’s (2009) scale of WFC* | | BAT-12 | | 1575 | | Logistic regression model | | 12 | | 56 | | Sweden | |  |
| Gynning et al. (2026)^7^ | WTFC | | Fisher’s (2009) scale of WFC* | | BAT-12 | | 4,132 | | Linear regression | | 12 | | 78 | | Sweden | | + |
| Hertzberg et al. (2016)^8^ | WTFC | | Cooper’s Job Stress Questionnaire | | OLBI | | 293 | | Linear regression model | | 60 | | 57 | | Norway | |  |
| Hu & He (2018)^9^ | WTFC | | QEEW | | MBI-GS* | | 445 | | Mediation analysis | | 12 | | 57 | | China Mainland | | + |
| Jaegers et al. (2021)^10^ | WTFC | | Unstandardised continuous measure | | Prison Social Climate Survey* | | 144 | | Mixed-effects model | | 1.5, 6, 12 | | 50 | | USA | | + |
| Jensen (2016)^11^ | WTFC | | Netemeyer’s (1996) scale of WFC* | | MBI-GS* | | 1703 | | Cross-lagged panel model | | 12 | | 23 | | Norway | | + |
| Jensen & Knudsen (2017)^12^ | WTFC | | Netemeyer’s (1996) scale of WFC* | | MBI-GS* | | 1702 | | Cross-lagged panel model | | 12 | | 12 | | Norway | | + |
| Junça Silva & Guarda (2026) ^13^ | WTFC | | Netemeyer’s (1996) scale of WFC* | | MBI-GS* | | 396 | | Mediation | | 0.5 | | 73 | | Portugal | | + |
| Leineweber et al. (2013)^13^ | WTFC | | Unstandardised continuous measure | | MBI-GS* | | 5938 | | Logistic regression model | | 24 | | 56 | | Sweden | |  |
| Lizano & Mor Barak (2012)^14^ | WTFC | | Beatty's (1996) scale of WFC | | MBI-GS | | 335 | | Growth curve analysis | | 6 | | 83 | | USA | |  |
| Richter et al. (2015)^15^ | WTFC | | Unstandardised continuous measure | | MBI-GS | | 3387 | | Cross-lagged panel model | | 24 | | 53 | | Sweden | |  |
| Shi et al. (2024)^16^ | WTFC | | Netemeyer’s (1996) scale of WFC* | | MBI-GS | | 324 | | Mediation analysis | | 0.07 | | 46 | | China Mainland | | + |
| Travis et al. (2016)^17^ | WTFC | | Beatty's (1996) scale of WFC | | MBI-GS | | 133 | | Mediation analysis | | 12 | | 83 | | USA | | + |
| vanHooff et al. (2005)^18^ | WTFC | | SWING | | MBI-GS* | | 730 | | Path analysis | | 12 | | 9 | | Netherlands | | + |
| Vieten et al. (2022)^19^ | WTFC | | Unstandardised continuous measure | | Oldenburg Burnout Inventory* | | 21308 | | Cross-lagged panel model | | 24 | | 48 | | Germany | | + |
| Westman et al. (2008)^20^ | WTFC | | Unstandardised continuous measure | | MBI-GS* | | 66 | | Linear regression analysis | | 0.1, 0.25, 0.5 | | 30 | | Israel | | + |
| Siu & Ng (2021)^21^ | FTWC | | Carlson’s (2000) scale of WFC* | | Unstandardised continuous measure | | 233 | | Mediation analysis | | 7 | | 64 | | Hong Kong | | + |
| Brzykcy et al. (2024)^22^ | WTFC & FTWC | | Netemeyer’s (1996) scale of WFC | | OLBI* | | 26133 | | Cross-lagged panel model | | 0.07, 0.25, 1, 6, 12 | | 47 | | Germany | | + |
| Innstrand et al. (2008)^23^ | WTFC & FTWC | | Wayne’s (2024) scale of WFC* | | OLBI | | 2235 | | Structural equation model | | 24 | | 46 | | Norway | | + |
| Innstrand et al. (2011)^24^ | WTFC & FTWC | | Unstandardised continuous measure | | OLBI | | 308 | | Linear regression analysis | | 24 | | 0 | | Norway | | + |
| Langballe et al. (2011)^25^ | WTFC & FTWC | | Wayne’s (2024) scale of WFC* | | OLBI | | 523 | | Linear regression analysis | | 24 | | 50 | | Norway | | + |
| Lee & Eissenstat (2018)^26^ | WTFC & FTWC | | Netemeyer’s (1996) scale of WFC | | MBI-GS | | 507 | | Growth model analysis | | 6 | | 45 | | USA | |  |
| Ren et al. (2025)^27^ | WTFC & FTWC | | Haslam’s (2015) Work-Family Conflict Scale | | Parental Burnout Assessment* | | 1002 | | Structural equation model | | 6 | | 50 | | China | | + |
| Depressive symptoms |  | |  | |  | |  | |  | |  | |  | |  | |  |
| Cao et al. (2023)^28^ | WTFC | | CWFQ* | | CES-D-20 | | 1187 | | Cross-lagged panel model | | 9, 21, 60, 24 | | 100 | | USA | | + |
| Cho et al. (2021)^29^ | WTFC | | Unstandardised continuous measure | | CES-D-8 | | 1598 | | Ordinary least squares model | | 24 | | 51 | | USA | | + |
| Costa et al. (2023)^5^ | WTFC | | Unstandardised continuous measure | | PHQ-9 | | 1843 | | Logistic regression model | | 12 | | 83 | | Portugal | |  |
| Goodman et al. (2009)^30^ | WTFC | | NWFSS | | CES-D | | 414 | | Structural equation model | | 9 | | 100 | | USA | | + |
| Hammer et al. (2005)^31^ | WTFC | | Netemeyer’s (1996) scale of WFC | | CES-D-20 | | 468 | | Cross-lagged panel model | | 12 | | 50 | | USA | |  |
| Jaegers et al. (2021)^10^ | WTFC | | Unstandardised continuous measure | | CES-D | | 144 | | Mixed-effects model | | 1.5, 6, 12 | | 50 | | USA | | + |
| Magnusson Hanson et al. (2014a)^32^ | WFTC | | Unstandardised continuous measure | | Hopkins Symptom Checklist-Depression | | 3224 | | Logistic regression model | | 24 | | 55 | | Sweden | |  |
| Nyberg et al. (2018)^33^ | WTFC | | Fisher’s (2009) scale of WFC* | | SCL-CD | | 1458 | | Cross-lagged panel model | | 24 | | 51 | | Sweden | | + |
| Song et al. (2024)^34^ | WTFC | | Unstandardised continuous measure | | CES-D-10 | | 5174 | | Linear regression model | | 96 | | 100 | | South Korea | | + |
| Steinmetz et al. (2008)^35^ | WTFC | | Netemeyer’s (1996) scale of WFC* | | Unstandardised continuous measure | | 130 | | Path analysis | | 12 | | 62 | | Germany | | + |
| Suh & Punnett (2020)^36^ | WTFC | | Work Interference with Family Scale* | | CES-D-10 | | 939 | | Logistic regression model | | 24 | | 80 | | USA | |  |
| Thorup et al. (2025)^37^ | WTFC | | COPSOQ-II | | CES-D-4 | | 679 | | Logistic regression model | | 48 | | 64.7 | | Denmark | |  |
| vanHooff et al. (2005)^18^ | WTFC | | SWING | | CES-D-8 | | 730 | | Path analysis | | 12 | | 9 | | Netherlands | | + |
| Bergs et al. (2018)^38^ | FTWC | | SWING* | | HAD* | | 2924 | | Cross-lagged structural equation model | | 24, 48 | | 19.5 | | Denmark | | + |
| Ju et al. (2018)^39^ | FTWC | | Unstandardised continuous measure | | CES-D-11 | | 4664 | | Logistic regression model | | 12 | | 100 | | South Korea | |  |
| Coursolle et al. (2010)^40^ | WTFC & FTWC | | Unstandardised continuous measure | | CES-D | | 2666 | | Ordinary least squares model | | 132 | | 45 | | USA | | + |
| Frone et al. (1997)^41^ | WTFC & FTWC | | Unstandardised continuous measure | | CES-D | | 267 | | Ordinary least squares model | | 48 | | 52 | | USA | | + |
| Grice et al. (2011)^42^ | WTFC & FTWC | | Unstandardised continuous measure | | The Short Form 12 | | 541 | | Linear regression analysis | | 1.25, 1.5, 4.5, 6 | | 100 | | USA | |  |
| Kayaalp et al. (2021)^43^ | WTFC & FTWC | | Kelloway’s (1999) scale of WFC | | CES-D-8 | | 1007 | | Cross-lagged panel model | | 12 | | 44 | | USA | | + |
| McTernan et al. (2016)^44^ | WTFC & FTWC | | Netemeyer’s (1996) scale of WFC* | | PHQ-9 | | 2793 | | Cross-lagged panel model | | 12 | | 52 | | Australia | | + |
| Peter et al. (2016)^45^ | WTFC & FTWC | | Copenhagen Psychosocial Questionnaire* | | BDI-V | | 3440 | | Cross-lagged panel model | | 48 | | 52 | | Germany | | + |
| Yucel et al. (2022)^46^ | WTFC & FTWC | | Unstandardised continuous measure | | STDS* | | 1262 | | Cross-lagged panel model | | 24, 48 | | 50 | | Germany | | + |
| General mental distress | |  | |  | |  | |  | |  | |  | |  | |  | |
| Caines & Treuren (2024)^47^ | WTFC | | Unstandardised continuous measure | | K10 | | 2867 | | Linear regression analysis | | 12 | | 50 | | Australia | |  |
| Kinnunen et al. (2004)^48^ | WTFC | | Stephens & Sommer’ (1996) scale of WFC | | GHQ-12 | | 406 | | Linear regression analysis | | 12 | | 100 | | Finland | | + |
| Laine et al. (2014)^49^ | WTFC | | Grzywacz & Marks’ Work-Family Interface Inventory | | GHQ-12 | | 3298 | | Logistic regression model | | 72 | | 80 | | Finland | |  |
| Marti et al. (2022)^50^ | WTFC | | Unstandardised continuous measure | | HSCL-25 | | 2382 | | Logistic regression model | | 28 | | 48 | | Norway | |  |
| Matthews et al. (1996)^51^ | WTFC | | Unstandardised continuous measure | | Unstandardised continuous measure | | 674 | | Structural equation model | | 12 | | 50 | | USA | |  |
| Neto et al. (2016)^52^ | WTFC | | Unstandardised continuous measure | | GHQ-12 | | 713 | | Structural equation model | | 6, 12 | | 18 | | Portugal | | + |
| Oshio et al. (2017a)^53^ | WTFC | | Unstandardised continuous measure | | K6 | | 7551 | | Linear probability models | | 12 | | 21 | | Japan | |  |
| Oshio et al. (2017b)^54^ | WTFC | | Unstandardised continuous measure | | K6 | | 7419 | | Logistic regression model | | 12 | | 21 | | Japan | |  |
| Petrie et al. (2023)^55^ | WTFC | | Unstandardised continuous measure | | K6 | | 383 | | Logistic regression model | | 12 | | 56 | | Australia | |  |
| Drummond et al. (2017)^56^ | WTFC & FTWC | | Carlson’s (2000) scale of WFC* | | GHQ-12 | | 2183 | | Path analysis | | 12 | | 76 | | Australia, New Zealand, China Mainland, Hong Kong | | + |
| Matthews et al. (2014)^57^ | WTFC & FTWC | | Fisher’s (2009) scale of WFC | | GHQ-12 | | 432 | | Cross-lagged panel model | | 1, 6 | | 69 | | USA | | + |
| Rantanen et al. (2008)^58^ | WTFC & FTWC | | Unstandardised continuous measure | | GHQ-12 | | 518 | | Correlation test | | 12 | | 50 | | Finland | | + |
| Vander Elst et al. (2026)^59^ | WTFC & FTWC | | QPSNordic* | | GHQ-12 | | 5959 | | Cross-lagged panel model | | 1 | | 70.3 | | Belgium | | + |
| Sun et al. (2021)^60^ | WTFC & FTWC | | Netemeyer’s (1996) scale of WFC | | GHQ-12 | | 351 | | Mediation analysis | | 0.7 | | 37 | | China Mainland | | + |
| Zu et al. (2020a)^61^ | WTFC & FTWC | | Unstandardised continuous measure | | GHQ-12 | | 220 | | Cross-lagged panel model | | 1.2 | | 59 | | China Mainland | | + |
| Zu et al. (2020b)^62^ | WTFC & FTWC | | Grzywacz & Marks’ Work-Family Interface Inventory | | GHQ-12 | | 220 | | Cross-lagged panel model | | 1.2 | | 59 | | China Mainland | | + |
| General mental well-being | |  | |  | |  | |  | |  | |  | |  | |  | |
| Grant-Vallone & Donaldson (2001)^63^ | WTFC | | Unstandardised continuous measure | | GWB* | | 342 | | Linear regression analysis | | 6 | | 70 | | USA | | + |
| Magee et al. (2012)^64^ | WTFC | | Work-Family Strains and Gains Scale* | | SF-36* | | 820 | | Cross-lagged panel model | | 12 | | 19 | | Australia | | + |
| Munir et al. (2012)^65^ | WTFC | | Copenhagen Psychosocial Questionnaire* | | Unstandardised continuous measure | | 188 | | Linear regression analysis | | 18 | | 93 | | Denmark | | + |
| Nguyen et al. (2016)^66^ | WTFC | | Work-Family Strains and Gains Scale* | | SF-36* | | 167 | | Linear regression analysis | | 24 | | 39 | | Australia | | + |
| Semerci et al. (2019)^67^ | WTFC | | Work-Family Strains and Gains Scale* | | SF-36* | | 866 | | Cross-lagged panel model | | 24 | | 43 | | Australia | | + |
| van der Heijden et al. (2008)^68^ | WTFC | | Netemeyer’s (1996) scale of WFC* | | SF-36* | | 1187 | | Cross-lagged panel model | | 12 | | 94 | | Netherlands | | + |
| Zhang et al. (2014)^69^ | WTFC | | Netemeyer’s (1996) scale of WFC | | PANAS | | 260 | | Cross-lagged panel model | | 1 | | 54 | | China Mainland | | + |
| Coursolle et al. (2010)^40^ | WTFC & FTWC | | Unstandardised continuous measure | | Positive well-being questionnaire | | 2855 | | Ordinary least squares model | | 132 | | 45 | | USA | | + |
| Reimann & Diewald (2022)^70^ | WTFC & FTWC | | Carlson’s (2000) scale of WFC | | SF-12 | | 4920 | | Linear regression model | | 24 | | 45 | | Germany | | + |
| Insomnia symptoms |  | |  | |  | |  | |  | |  | |  | |  | |  |
| Magnusson Hanson et al. (2014b)^71^ | WTFC | | Unstandardised continuous measure | | Karolinska Sleepiness Scale | | 3224 | | Logistic regression model | | 24 | | 55 | | Sweden | |  |
| Mäkelä et al. (2014)^72^ | WTFC | | Carlson’s (2000) scale of WFC* | | Unstandardised continuous measure | | 868 | | Mediation analysis | | 12 | | 76 | | Finland | | + |
| Antino et al. (2022)^73^ | WTFC & FTWC | | SWING | | Insomnia Severity Index | | 1519 | | Discontinuous growth models | | 0.21, 0.25 | | 76 | | Spain | | + |
| Vedaa et al. (2016)^74^ | WTFC & FTWC | | Unstandardised continuous measure | | Bergen Insomnia Scale | | 799 | | Structural equation model | | 24 | | 90 | | Norway | | + |
| Vleeshouwers et al. (2019)^75^ | WTFC & FTWC | | QPSNordic* | | Unstandardised continuous measure | | 4681 | | Mediation | | 24 | | 55.1 | | Norway | |  |
| Problem drinking |  | |  | |  | |  | |  | |  | |  | |  | |  |
| Leineweber et al. (2013)^13^ | WTFC | | Unstandardised continuous measure | | CAGE Alcohol scale | | 5669 | | Logistic regression model | | 24 | | 56 | | Sweden | |  |
| Oshio et al. (2017a)^53^ | WTFC | | Unstandardised continuous measure | | Unstandardised continuous measure | | 7551 | | Linear probability models | | 12 | | 21 | | Japan | |  |
| Frone et al. (1997)^41^ | WTFC & FTWC | | Unstandardised continuous measure | | Unstandardised continuous measure | | 267 | | Ordinary least squares model | | 48 | | 52 | | USA | |  |
| Maekubo et al. (2025)^76^ | WTFC & FTWC | | SWING | | AUDIT | | 640 | | Logistic regression model | | 6 | | 29.5 | | Japan | |  |
| Wolff et al. (2014)^77^ | WTFC & FTWC | | Kelloway’s (1999) scale of WFC | | Unstandardised continuous measure | | 543 | | Linear regression analysis | | 12 | | 51 | | USA | |  |
| General stress |  | |  | |  | |  | |  | |  | |  | |  | |  |
| Thorup et al. (2025)^37^ | WTFC | | COPSOQ-II | | PSS | | 679 | | Logistic regression model | | 48 | | 64.7 | | Denmark | |  |
| Brzykcy et al. (2024)^22^ | WTFC & FTWC | | Netemeyer’s (1996) scale of WFC | | Unstandardised continuous measure | | 26133 | | Cross-lagged panel model | | 0.07, 0.25, 1, 6, 12 | | 47 | | Germany | |  |
| Kelloway et al. (1999)^78^ | WTFC & FTWC | | Kelloway’s (1999) scale of WFC | | Unstandardised continuous measure | | 263 | | Structural equation model | | 6 | | 69 | | Canada | |  |
| Smoktunowicz & Cieslak (2018)^79^ | WTFC & FTWC | | Netemeyer’s (1996) scale of WFC | | PSS | | 260 | | Structural equation model | | 3 | | 50 | | Poland | |  |
| Weale et al. (2023)^80^ | WTFC & FTWC | | Copenhagen Psychosocial Questionnaire* | | Copenhagen Psychosocial Questionnaire* | | 269 | | Mediation analysis | | 6 | | 80 | | Australia | |  |
| Anxiety symptoms |  | |  | |  | |  | |  | |  | |  | |  | |  |
| Costa et al. (2023)^5^ | WTFC | | Unstandardised continuous measure | | GAD-7 | | 1843 | | Logistic regression model | | 12 | | 83 | | Portugal | |  |
| Kayaalp et al. (2021)^43^ | WTFC & FTWC | | Kelloway’s (1999) scale of WFC | | POMS* | | 1007 | | Cross-lagged panel model | | 12 | | 44 | | USA | |  |
| Parenting stress |  | |  | |  | |  | |  | |  | |  | |  | |  |
| Kinnunen et al. (2004)^48^ | WTFC | | Stephens & Sommer’ (1996) scale of WFC | | Parenting Stress Index* | | 206 | | Linear regression analysis | | 12 | | 100 | | Finland | |  |
| Kinnunen et al. (2010)^81^ | WTFC & FTWC | | Stephens & Sommer’ (1996) scale of WFC | | Parenting Stress Index* | | 478 | | Structural equation model | | 12 | | 50 | | Finland | |  |
| Major Depressive Disorder diagnosis | |  | |  | |  | |  | |  | |  | |  | |  | |
| Wang et al. (2012a)^82^ | WTFC & FTWC | | Unstandardised continuous measure | | Diagnostic Interview | | 2572 | | Logistic regression model | | 12 | | 44 | | Canada | |  |
| Wang et al. (2012b)^83^ | WTFC & FTWC | | Unstandardised continuous measure | | Diagnostic Interview | | 661 | | Logistic regression model | | 12 | | 59 | | Canada | |  |
| PTSD symptoms |  | |  | |  | |  | |  | |  | |  | |  | |  |
| Costa et al. (2023)^5^ | WTFC | | Unstandardised continuous measure | | PCL-5 | | 1843 | | Logistic regression model | | 12 | | 83 | | Portugal | |  |
| Combined depression & anxiety | |  | |  | |  | |  | |  | |  | |  | |  | |
| Antino et al. (2022)^73^ | WTFC & FTWC | | SWING | | PHQ-4 | | 1519 | | Linear regression model | | 0.21, 0.25 | | 76 | | Spain | |  |
| Common mental disorders diagnosis | |  | |  | |  | |  | |  | |  | |  | |  | |
| Razavi et al. (2015)^84^ | WTFC | | Unstandardised continuous measure | | CIS-R | | 9008 | | Logistic regression model | | 36 | | 50 | | UK | |  |
| Having treatment of antidepressant |  | |  | |  | |  | |  | |  | |  | |  | |  |
| Magnusson Hanson et al. (2014a)^32^ | WTFC | | Unstandardised continuous measure | | Binary self-report | | 3224 | | Cox proportional hazards regression model | | 24 | | 55 | | Sweden | |  |

**Note:** * Used individual or modified items or sub-scales rather than the complete measurement; † Multiple numbers indicate that the study tested the associations in different follow-up intervals.

Abbreviations: WTFC= Work-to-family conflict; FTWC= Family-to-work conflict; PTSD= Post-traumatic stress disorder; WFBS=Work-Family Balance Scale; QEEW= Questionnaire on Experience and Evaluation of Work; SWING= the Survey Work–home Interaction-NijmeGen; QPSNordic=The general Nordic questionnaire; CWFQ=Combining Work and Family Questionnaire; NWFSS=Negative Work-Family Spillover Scale; COPSOQ-II= Copenhagen Psychosocial Questionnaire II; HAD=Hospital Anxiety and Depression scale; CES-D=Centre for Epidemiologic Studies Depression; GWB=General Well-Being Schedule; GHQ-12=General Health Questionnaire; MBI-GS=Maslach Burnout Inventory-General Survey; POMS=Profile of Mood States; SF-36=36-Item Short-Form Health Survey; PHQ-9=Patient Health Questionnaire-9; SCL-CD=Symptom Checklist-Core Depression Scale; K6=6-Item Kessler’s scale; K10=10-Item Kessler’s scale; BDI=Beck-Depressions Inventory; STDS=State-Trait Depression Scale; PANAS= Positive Affect and Negative Affect Scale; CIS-R=Revised Clinical Interview Schedule; HSCL-25 = Hopkins Symptoms Checklist-25; SMBM= Shirom–Melamed Burnout Measure; PCL-5= Short Form of the Post-traumatic Stress Disorder Checklist; AUDIT=Alcohol Use Disorders Identification Test; PSS=Perceived Stress Scale; GAD-7=Generalised Anxiety Disorder Scale-7; OLBI=Oldenburg Burnout Inventory; BMS=Burnout Measure-Short Version; BAT-12= Burnout Assessment Tool 12.

| **Table E. Characteristics of studies reporting logistic regression of longitudinal association between work-family conflict and mental health outcomes** | | | | |
| --- | --- | --- | --- | --- |
| **Study** | **Exposure** | **Exposure modality** | **Outcome** | **Results** |
| **Costa et al. (2023)**^5^ | WTFC | Binary (Yes; No) | Burnout | Significant positive associations for all four outcomes |
|  |  |  | Depressive symptoms |  |
|  |  |  | Anxiety symptoms |  |
|  |  |  | PTSD symptoms |  |
| **Gynning et al. (2024)**^6^ | WTFC | Continuous score | Burnout | Significant positive associations |
| **Leineweber et al. (2013)**^13^ | WTFC | Three-level categorical  (Low; Moderate; High) | Burnout | Significant positive associations for both outcomes |
|  |  |  | Problem drinking |  |
| **Magnusson Hanson et al. (2014a)**^32^ | WTFC | Three-level categorical  (Low; Moderate; High) | Depressive symptoms | Significant positive associations |
| **Suh & Punnett (2020)**^36^ | WTFC | Binary (High; Low) | Depressive symptoms | Significant positive associations |
| **Thorup et al. (2025)**^37^ | WTFC | Continuous | Depressive symptoms | Significant and non-significant positive associations |
| **Ju et al. (2018)**^39^ | FTWC | Binary (Yes; No) | Depressive symptoms | Significant positive associations |
| **Laine et al. (2014)**^49^ | WTFC | Three-level categorical  (No; Weak; Strong) | General mental distress | Significant positive associations |
| **Marti et al. (2022)**^50^ | WTFC | Three-level categorical  (No; Occasional; Frequent) | General mental distress | Non-significant association |
| **Oshio et al. (2017b)**^54^ | WTFC | Binary (High; Low) | General mental distress | Significant positive associations |
| **Petrie et al. (2023)**^55^ | WTFC | Binary (Yes; No) | General mental distress | Significant positive associations |
| **Magnusson Hanson et al. (2014b)**^71^ | WFTC | Continuous | Insomnia | Significant positive associations |
| **Maekubo et al. (2025)**^76^ | WTFC & FTWC | Continuous | Problem drinking | Significant positive associations for WTFC, non-significant for FTWC |
| **Wang et al. (2012a)**^82^ | WTFC & FTWC | Binary (High; Low) | Major Depressive Disorder diagnosis | Significant positive associations |
| **Wang et al. (2012b)**^83^ | WTFC & FTWC | Binary (High; Low) | Major Depressive Disorder diagnosis | Significant positive associations |
| **Razavi et al. (2015)**^84^ | WTFC | Binary (Yes; No) | Common mental disorder diagnosis | Significant positive associations |

**Note:** WTFC= Work-to-family conflict; FTWC= Family-to-work conflict; PTSD= Post-traumatic stress disorder

# **Table F. Confounding adjustment conditions of the articles included in meta-analysis**

| **Study ID** | **Confounding Adjustment Conditions *** | **Specific covariates adjusted ^†^** |
| --- | --- | --- |
| Studies involved in synthesisation of effect sizes with comprehensive confounding adjustment | | |
| Cao et al. (2023) | Demo_Work_Family_Baseline | Sex (stratification), age, education, ethnicity, child sex, maternal work commitment, maternal neuroticism, child-temperament, family income-to-need ratio, baseline mental health |
| Cho et al. (2021) | Demo_Work_Family_Baseline | Age, sex, race, education, marital status, income and wealth; Employment status; Self-rated health at baseline, disability at baseline, chronic conditions at baseline, depressive symptoms at baseline, physical activities, baseline mental health |
| Coursolle et al. (2010) | Demo_Work_Family_Baseline | Gender, wages, assets, physical health, and educational attainment, marital context, duration of the current marriage, spousal employment status, spousal health status, caregiving responsibilities, and whether children were living in the household, long working hours, class of worker, concentration requirement of work, exposed the respondent to dangerous conditions, required work under time pressure, general job satisfaction, availability of pension plans or health insurance, baseline mental health |
| Drummond et al. (2017) | Demo_Work_Family_Baseline | Gender (biological sex), age, marital status, educational qualifications, number of dependants currently living with respondents, supervisor support, baseline support, baseline mental health |
| Grant-Vallone & Donaldson (2001) | Demo_Work_Family_Baseline | Gender, marital status, number of children, working hour, baseline mental health |
| Innstrand et al. (2011) | Demo_Work_Family_Baseline | Age, the prevalence of young children (below the age of six years), years of education, and number of hours worked per week, baseline mental health |
| Kayaalp et al. (2021) | Demo_Work_Family_Baseline | Gender, age, ethnicity, household income, hours worked per week, number of care-recipients, whether care recipients had a disability or illness, baseline mental health |
| Kinnunen et al. (2004) | Demo_Work_Family_Baseline | Age, sex (stratified), socio-economic status, working hours/week, working schedule, living with partner, number of children living at home, baseline mental health |
| Langballe et al. (2011) | Demo_Work_Family_Baseline | Age, sex (stratified) working hours, marital status, number of children under the age of 6 years, baseline mental health |
| Mäkelä et al. (2014) | Demo_Work_Family_Baseline | Gender, age, relationship status (yes/no), children (yes/no), body mass index, alcohol consumption (units/week), smoking currently (yes/no), smoking previously (yes/no), physical exercise, holding a supervisory position (yes/no), workload, baseline mental health. |
| Nyberg et al. (2018) | Demo_Work_Family_Baseline | Age, labour market sector, education, marital status, child living at home, turn over, baseline mental health |
| Siu & Ng (2021) | Demo_Work_Family_Baseline | Age, gender, education, position, shift, tenure, marital status, child living at home, work engagement, safety violations; workplace injuries |
| Vieten et al. (2022) | Demo_Work_Family_Baseline | Gender, age, education level, living with a partner, underage child in household, weekly working hours, regular day work, requirement levels according to the German classification of occupations (KldB 2010), baseline mental health |
| Yucel et al. (2022) | Demo_Work_Family_Baseline | Age, sex (stratified), education, work hours, presence of a preschool child living in the household, baseline mental health |
| Bergs et al. (2018) | Demo_Work_Baseline | Gender, age, work schedule, baseline mental health |
| Brzykcy et al. (2024) | Demo_Work_Baseline | Baseline mental health; time-invariant confounders (gender, age, education, occupation type) |
| Gynning et al. (2026) | Demo_Work_Baseline | Sex, age, birth country, years of working experience, working hours, county of work, baseline mental health |
| Peter et al. (2016) | Demo_Work_Baseline | Age, sex (stratified), occupational position, negative affectivity, work over-commitment, (un)employed episodes, region, baseline mental health. |
| Vander Elst et al. (2026) | Demo_Work_Baseline | Telecommuting, social support, baseline mental health; time-invariant confounders (gender, age, education), baseline mental health |
| vanHooff et al. (2005) | Demo_Work_Baseline | Gender, age, workload, job control, baseline mental health |
| Studies only involved in overall synthesisation | | |
| Magee et al. (2012) | Demo_Family_Baseline | Age, sex, number of children, partner, employment status, baseline mental health |
| Matthews et al. (2014) | Demo_Family_Baseline | Age, gender, child-care responsibilities, baseline mental health |
| Neto et al. (2016) | Demo_Family_Baseline | Sex, age, number and age of children (parental demand), baseline mental health |
| Ren et al. (2025) | Demo_Family_Baseline | Age, sex (stratified), child's age, child's gender, family fertility situation, baseline mental health, partner's work-family conflict, baseline mental health |
| Chow et al. (2024) | Demo_Work_Family | Gender, age, education level, annual household income, employment status, weekly working hours, marriage status, years in marriage, having children |
| Munir et al. (2012) | Demo_Work_Family | Age, gender, length of employment (tenure), partner and children living at home |
| Nguyen et al. (2016) | Demo_Work_Family | Gender, highest education level, occupational tenure, business income, marital status, children under age of 14, age, |
| Song et al. (2024) | Demo_Work_Family | Age, sex (stratified), years of education, marital duration, number of births, household size, household income (log-transformed), and occupation type (managerial, service, and labour occupations) |
| Sun et al. (2021) | Demo_Work_Family | Sex, age, education, marriage, monthly income, Job position, level, working overtime |
| Antino et al. (2022) | Demo_Work | Age, educational level, risky job category, number of children, number of dependent persons in charge, professional sector, having a garden/terrace during quarantine, nationality or region, COVID-19 experience |
| Baka & prusik (2021) | Demo_Work | Gender, age, education, seniority at work, quantitative demands, work-pace, cognitive demands, emotional demands, demands for hiding emotions |
| Travis et al. (2016) | Demo_Work | Age, gender (male/female) and organisational tenure |
| van der Heijden et al. (2008) | Demo_Work | Socio-demographics (unspecified), Job demands |
| Westman et al. (2008) | Demo_Work | Gender, perceived control, social support, and number of travels |
| Zu et al. (2020b) | Demo_Work | Gender, education, job experience |
| Goodman et al. (2009) | Demo_Family | Sex (constant) age, education, race, and partner status, baseline mental health |
| Frone et al. (1997) | Demo_Baseline | Gender, race, age (in years), education (in years), family income, baseline mental health |
| Jensen (2016) | Work_Baseline | Work-role conflict, baseline mental health |
| Jensen & Knudsen (2017) | Work_Baseline | Business travel, baseline mental health |
| McTernan et al. (2016) | Work_Baseline | Co-worker support, mining/unmining, baseline mental health |
| Junça Silva & Guarda (2026) | Demo | Age and sex |
| Shi et al. (2024) | Work | Work-place fear of missing out, work-related psychological detachment |
| Rantanen et al. (2008) | Baseline | Baseline mental health |
| Reimann & Diewald (2022) | Baseline | Baseline mental health |
| Zhang et al. (2014) | Baseline | Baseline mental health |
| Hu & He (2018) | Unknown | Unknown |
| Innstrand et al. (2008) | Unknown | Unknown |
| Jaegers et al. (2021) | Unknown | Unknown |
| Steinmetz et al. (2008) | Unknown | Unknown |
| Vedaa et al. (2016) | Unknown | Unknown |
| Semerci et al. (2019) | Unknown | Unknown |
| Zu et al. (2020a) | Unknown | Unknown |

**Note:** *One study could have multiple effect sizes with varying confounding adjustment conditions, here we report the most comprehensively adjusted effect size in each study; † Original wording from each paper; Demo=sociodemographic variables at individual level; Work= work-related demands and resources; Family=family-related demands and resources; Baseline=baseline mental health; Unknown=not mentioning any confounding adjustment.

| **Table G. Quality check for the articles included in meta-analysis** | | | | | | | | | | | | | |
| --- | --- | --- | --- | --- | --- | --- | --- | --- | --- | --- | --- | --- | --- |
| **Study ID** | **Bias from selection** | | | | **Bias from Comparability** | | **Bias from Outcome** | | | **Scoring** | | | **Overall Quality*** |
|  | **Sample representativeness** | **Non-exposed participants selection** | **Ascertainment of exposure** | **Control for baseline outcome** | **Control for basic demographics** | **Control for other factors** | **Outcome assessment** | **Adequate follow-up length** | **Adequate follow-up cohort** | **Selection** | **Comparability** | **Outcome** |  |
| Bergs et al. (2018) | Truly or somewhat representative of working population | Drawn from the same source | Written self-report | Yes | Yes | Yes | Self-report | Yes (>1 months) | Subjects lost to follow up unlikely to introduce bias | 3 | 2 | 2 | Good |
| Brzykcy et al. (2024) | Truly or somewhat representative of working population | Drawn from the same source | Written self-report | Yes | Yes | Yes | Self-report | Yes (>1 months) | Subjects lost to follow up unlikely to introduce bias | 3 | 2 | 2 | Good |
| Cao et al. (2023) | Truly or somewhat representative of working population | Drawn from the same source | Interviews | Yes | Yes | Yes | Self-report | Yes (>1 months) | Subjects lost to follow up unlikely to introduce bias | 4 | 2 | 2 | Good |
| Cho et al. (2021) | Truly or somewhat representative of working population | Drawn from the same source | Written self-report | Yes | Yes | Yes | Self-report | Yes (>1 months) | Subjects lost to follow up unlikely to introduce bias | 3 | 2 | 2 | Good |
| Coursolle et al. (2010) | Truly or somewhat representative of working population | Drawn from the same source | Interview OR written self-report | Yes | Yes | Yes | Self-report | Yes (>1 months) | Subjects lost to follow up unlikely to introduce bias | 3 | 2 | 2 | Good |
| Frone et al. (1997) | Truly or somewhat representative of working population | Drawn from the same source | Interview | Yes | Yes | Yes | Self-report | Yes (>1 months) | Subjects lost to follow up unlikely to introduce bias | 4 | 2 | 2 | Good |
| Gynning et al. (2026) | Truly or somewhat representative of working population | Drawn from the same source | Interview OR written self-report | Yes | Yes | Yes | Self-report | Yes (>1 months) | Subjects lost to follow up unlikely to introduce bias | 3 | 2 | 2 | Good |
| Goodman et al. (2009) | Selected group | Drawn from the same source | Interview | Yes | Yes | Yes | Self-report | Yes (>1 months) | Subjects lost to follow up unlikely to introduce bias | 3 | 2 | 2 | Good |
| Kayaalp et al. (2021) | Truly or somewhat representative of working population | Drawn from the same source | Written self-report | Yes | Yes | Yes | Self-report | Yes (>1 months) | Subjects lost to follow up unlikely to introduce bias | 3 | 2 | 2 | Good |
| Kinnunen et al. (2004) | Truly or somewhat representative of working population | Drawn from the same source | Written self-report | Yes | Yes | Yes | Self-report | Yes (>1 months) | Subjects lost to follow up unlikely to introduce bias | 3 | 2 | 2 | Good |
| Magee et al. (2012) | Truly or somewhat representative of working population | Drawn from the same source | Interviews | Yes | Yes | Yes | Self-report | Yes (>1 months) | Subjects lost to follow up unlikely to introduce bias | 4 | 2 | 2 | Good |
| Matthews et al. (2014) | Truly or somewhat representative of working population | Drawn from the same source | Written self-report | Yes | Yes | Yes | Self-report | Yes (>1 months) | Subjects lost to follow up unlikely to introduce bias | 3 | 2 | 2 | Good |
| McTernan et al. (2016) | Truly or somewhat representative of working population | Drawn from the same source | Interviews | Yes | No | Yes | Self-report | Yes (>1 months) | Complete follow up-all subjects accounted | 4 | 1 | 2 | Good |
| Rantanen et al. (2008) | Truly or somewhat representative of working population | Drawn from the same source | Written self-report | Yes | No | Yes | Self-report | Yes (>1 months) | Subjects lost to follow up unlikely to introduce bias | 3 | 1 | 2 | Good |
| Vander Elst et al. (2026) | Truly or somewhat representative of working population | Drawn from the same source | Written self-report | Yes | Yes | Yes | Self-report | Yes (>1 months) | Subjects lost to follow up unlikely to introduce bias | 3 | 2 | 2 | Good |
| Vieten et al. (2022) | Truly or somewhat representative of working population | Drawn from the same source | Interviews | Yes | Yes | Yes | Self-report | Yes (>1 months) | Complete follow up-all subjects accounted | 4 | 2 | 2 | Good |
| Yucel et al. (2022) | Truly or somewhat representative of working population | Drawn from the same source | Interviews | Yes | Yes | Yes | Self-report | Yes (>1 months) | Complete follow up-all subjects accounted | 4 | 2 | 2 | Good |
| Grant-Vallone & Donaldson (2001) | Selected group | Drawn from the same source | Written self-report | Yes | Yes | Yes | Self-report & Other report | Yes (>1 months) | Subjects lost to follow up unlikely to introduce bias | 2 | 2 | 2 | Fair |
| Jensen (2016) | Selected group | Drawn from the same source | Written self-report | Yes | No | Yes | Self-report | Yes (>1 months) | Subjects lost to follow up unlikely to introduce bias | 2 | 1 | 2 | Fair |
| Jensen & Knudsen (2017) | Selected group | Drawn from the same source | Written self-report | Yes | No | Yes | Self-report | Yes (>1 months) | Subjects lost to follow up unlikely to introduce bias | 2 | 1 | 2 | Fair |
| Mäkelä et al. (2014) | Selected group | Drawn from the same source | Written self-report | Yes | Yes | Yes | Self-report | Yes (>1 months) | Subjects lost to follow up unlikely to introduce bias | 2 | 2 | 2 | Fair |
| Neto et al. (2016) | Selected group | Drawn from the same source | Written self-report | Yes | Yes | Yes | Self-report | Yes (>1 months) | Subjects lost to follow up unlikely to introduce bias | 2 | 2 | 2 | Fair |
| Reimann & Diewald (2022) | Selected group | Drawn from the same source | Interviews | No | Yes | Yes | Self-report | Yes (>1 months) | Subjects lost to follow up unlikely to introduce bias | 2 | 2 | 2 | Fair |
| Ren et al. (2025) | Selected group | Drawn from the same source | Written self-report | Yes | Yes | Yes | Self-report | Yes (>1 months) | Subjects lost to follow up unlikely to introduce bias | 2 | 2 | 2 | Fair |
| Siu & Ng (2021) | Selected group | Drawn from the same source | Written self-report | Yes | Yes | Yes | Self-report | Yes (>1 months) | Subjects lost to follow up unlikely to introduce bias | 2 | 2 | 2 | Fair |
| Song et al. (2024) | Truly or somewhat representative of working population | Drawn from the same source | Written self-report | No | Yes | Yes | Self-report | Yes (>1 months) | Subjects lost to follow up unlikely to introduce bias | 2 | 2 | 2 | Fair |
| vanHooff et al. (2005) | Selected group | Drawn from the same source | Written self-report | Yes | Yes | Yes | Self-report | Yes (>1 months) | Subjects lost to follow up unlikely to introduce bias | 2 | 2 | 2 | Fair |
| Zu et al. (2020b) | Truly or somewhat representative of working population | Drawn from the same source | Written self-report | No | Yes | Yes | Self-report | Yes (>1 months) | Complete follow up-all subjects accounted | 2 | 2 | 2 | Fair |
| Antino et al. (2022) | Selected group | Drawn from the same source | Written self-report | No | Yes | Yes | Self-report | No | Subjects lost to follow up unlikely to introduce bias | 1 | 2 | 1 | Poor |
| Baka & prusik (2021) | Selected group | Drawn from the same source | Written self-report | No | Yes | Yes | Self-report | Yes (>1 months) | Follow up rate less than 80% and no description of those lost | 1 | 2 | 1 | Poor |
| Chow et al. (2024) | Truly or somewhat representative of working population | Drawn from the same source | Written self-report | No | Yes | Yes | Self-report | Yes (>1 months) | Subjects lost to follow up likely to introduce bias | 2 | 2 | 1 | Poor |
| Drummond et al. (2017) | Selected group | Drawn from the same source | Written self-report | No | Yes | Yes | Self-report | Yes (>1 months) | No statement | 1 | 2 | 1 | Poor |
| Hu & He (2018) | Selected group | Drawn from the same source | Written self-report | No | No | Yes | Self-report | Yes (>1 months) | No statement | 1 | 1 | 1 | Poor |
| Innstrand et al. (2008) | Truly or somewhat representative of working population | Drawn from the same source | Written self-report | No | Yes | Yes | Self-report | Yes (>1 months) | Follow up rate less than 80% and no description of those lost | 2 | 2 | 1 | Poor |
| Innstrand et al. (2011) | Selected group | Drawn from the same source | Written self-report | No | Yes | Yes | Self-report | Yes (>1 months) | Follow up rate less than 80% and no description of those lost | 1 | 2 | 1 | Poor |
| Jaegers et al. (2021) | Selected group | Drawn from the same source | Written self-report | No | Yes | Yes | Self-report | Yes (>1 months) | Complete follow up-all subjects accounted | 1 | 2 | 2 | Poor |
| Junça Silva & Guarda (2026) | Selected group | Drawn from the same source | Written self-report | No | Yes | No | Self-report | No | Subjects lost to follow up unlikely to introduce bias | 1 | 1 | 1 | Poor |
| Munir et al. (2012) | Selected group | Drawn from the same source | Written self-report | No | Yes | Yes | Self-report | Yes (>1 months) | Complete follow up-all subjects accounted | 1 | 2 | 2 | Poor |
| Langballe et al. (2011) | Truly or somewhat representative of working population | Drawn from the same source | Written self-report | Yes | Yes | Yes | Self-report | Yes (>1 months) | Follow up rate less than 80% and no description of those lost | 3 | 2 | 1 | Poor |
| Nguyen et al. (2016) | Selected group | Drawn from the same source | Interviews | No | Yes | Yes | Self-report | Yes (>1 months) | No statement | 2 | 2 | 1 | Poor |
| Nyberg et al. (2018) | Selected group | Drawn from the same source | Written self-report | Yes | Yes | Yes | Self-report | Yes (>1 months) | Follow up rate less than 80% and no description of those lost | 2 | 2 | 1 | Poor |
| Peter et al. (2016) | Truly or somewhat representative of working population | Drawn from the same source | Interviews + Record linkage | Yes | Yes | Yes | Self-report | Yes (>1 months) | Follow up rate less than 80% and no description of those lost | 4 | 2 | 1 | Poor |
| Semerci et al. (2019) | Truly or somewhat representative of working population | Drawn from the same source | Interviews | Yes | No | Yes | Self-report | Yes (>1 months) | Follow up rate less than 80% and no description of those lost | 4 | 1 | 1 | Poor |
| Shi et al. (2024) | Selected group | Drawn from the same source | Written self-report | No | Yes | Yes | Self-report | No | Subjects lost to follow up unlikely to introduce bias | 1 | 2 | 1 | Poor |
| Steinmetz et al. (2008) | Selected group | Drawn from the same source | Written self-report | No | No | No | Self-report | Yes (>1 months) | Subjects lost to follow up likely to introduce bias | 1 | 0 | 2 | Poor |
| Sun et al. (2021) | Truly or somewhat representative of working population | Drawn from the same source | Written self-report | No | Yes | Yes | Self-report | No (<1 month) | No statement | 2 | 2 | 0 | Poor |
| Travis et al. (2016) | Selected group | Drawn from the same source | Written self-report | No | Yes | Yes | Self-report | Yes (>1 months) | Subjects lost to follow up unlikely to introduce bias | 1 | 2 | 2 | Poor |
| van der Heijden et al. (2008) | Selected group | Drawn from the same source | Written self-report | No | Yes | Yes | Self-report | Yes (>1 months) | Follow up rate less than 80% and no description of those lost | 1 | 2 | 1 | Poor |
| Vedaa et al. (2016) | Selected group | Drawn from the same source | Written self-report | Yes | No | Yes | Self-report | Yes (>1 months) | Follow up rate less than 80% and no description of those lost | 2 | 1 | 1 | Poor |
| Westman et al. (2008) | Selected group | Drawn from the same source | Written self-report | No | Yes | Yes | Self-report | No (<1 month) | Subjects lost to follow up unlikely to introduce bias | 1 | 2 | 1 | Poor |
| Zhang et al. (2014) | Selected group | Drawn from the same source | Written self-report | Yes | No | No | Self-report | Yes (>1 months) | Complete follow up-all subjects accounted | 2 | 0 | 2 | Poor |
| Zu et al. (2020a) | Truly or somewhat representative of working population | Drawn from the same source | Interview & Written self-report | No | Yes | Yes | Self-report | Yes (>1 months) | No statement | 2 | 2 | 1 | Poor |

**Note:** *The classification was based on the criteria proposed by Newcastle-Ottawa quality assessment scale for cohort studies .

| **Table H. Sensitivity test of meta-analysis results through removing one study each time** | | | | |
| --- | --- | --- | --- | --- |
|  | **Pooled β** | **Pooled SE** | ***p*-value** | **Study**  **Quality** |
| **Work-to-family conflict and depressive symptoms** |  |  |  |  |
| All Studies | 0.11 | 0.02 | <0.001 |  |
| -Cao et al. (2023) | 0.11 | 0.02 | <0.001 | Good |
| -Cho et al. (2021) | 0.09 | 0.01 | <0.001 | Good |
| -Coursolle et al. (2010) | 0.11 | 0.02 | <0.001 | Good |
| -Goodman et al. (2009) | 0.10 | 0.02 | <0.001 | Good |
| -Kayaalp et al. (2021) | 0.11 | 0.02 | <0.001 | Good |
| -McTernan et al. (2016) | 0.12 | 0.02 | <0.001 | Good |
| -Yucel et al. (2022) | 0.12 | 0.02 | <0.001 | Good |
| -Song et al. (2024) | 0.11 | 0.02 | <0.001 | Fair |
| -vanHooff et al. (2005) | 0.11 | 0.02 | <0.001 | Fair |
| -Jaegers et al. (2021) | 0.11 | 0.02 | <0.001 | Poor |
| -Nyberg et al. (2018) | 0.11 | 0.02 | <0.001 | Poor |
| -Peter et al. (2016) | 0.11 | 0.02 | <0.001 | Poor |
| -Steinmetz et al. (2008) | 0.10 | 0.02 | <0.001 | Poor |
| **Work-to-family conflict and burnout** |  |  |  |  |
| All Studies | 0.20 | 0.04 | <0.001 |  |
| -Brzykcy et al. (2024) | 0.21 | 0.04 | <0.001 | Good |
| -Gynning et al. (2026) | 0.20 | 0.04 | <0.001 | Good |
| -Vieten et al. (2022) | 0.21 | 0.04 | <0.001 | Good |
| -Jensen (2016) | 0.21 | 0.04 | <0.001 | Fair |
| -Jensen & knudsen (2017) | 0.21 | 0.04 | <0.001 | Fair |
| -Ren et al. (2025) | 0.21 | 0.04 | <0.001 | Fair |
| -vanHooff et al. (2005) | 0.21 | 0.04 | <0.001 | Fair |
| -Baka & Prusik (2021) | 0.21 | 0.04 | <0.001 | Poor |
| -Chow et al. (2024) | 0.19 | 0.04 | <0.001 | Poor |
| -Hu & He (2018) | 0.19 | 0.04 | <0.001 | Poor |
| -Innstrand et al. (2008) | 0.21 | 0.04 | <0.001 | Poor |
| -Innstrand et al. (2011) | 0.22 | 0.04 | <0.001 | Poor |
| -Jaegers et al. (2021) | 0.21 | 0.04 | <0.001 | Poor |
| -Junça Silva & Guarda (2026) | 0.18 | 0.04 | <0.001 | Poor |
| -Langballe et al. (2011) | 0.21 | 0.04 | <0.001 | Poor |
| -Shi et al. (2024) | 0.20 | 0.04 | <0.001 | Poor |
| -Travis et al. (2016) | 0.20 | 0.04 | <0.001 | Poor |
| -Westman et al. (2008) | 0.20 | 0.04 | <0.001 | Poor |
| **Work-to-family conflict and general mental wellbeing** |  |  |  |  |
| All Studies | -0.10 | 0.01 | <0.001 |  |
| -Coursolle et al. (2010) | -0.10 | 0.01 | <0.001 | Good |
| -Magee et al. (2012) | -0.11 | 0.02 | <0.001 | Good |
| -Grant-Vallone & Donaldson (2001) | -0.09 | 0.01 | <0.001 | Fair |
| -Reimann & Diewald (2022) | -0.11 | 0.02 | 0.001 | Fair |
| -Munir et al. (2012) | -0.09 | 0.01 | <0.001 | Poor |
| -Nguyen et al. (2016) | -0.09 | 0.01 | <0.001 | Poor |
| -Semerci et al. (2019) | -0.10 | 0.02 | <0.001 | Poor |
| -van der Heijden et al. (2008) | -0.09 | 0.01 | <0.001 | Poor |
| -Zhang et al. (2014) | -0.09 | 0.01 | <0.001 | Poor |
| **Work-to-family conflict and general mental distress** |  |  |  |  |
| All Studies | 0.15 | 0.03 | <0.001 |  |
| -Kinnunen et al. (2004) | 0.11 | 0.02 | <0.001 | Good |
| -Matthews et al. (2014) | 0.16 | 0.03 | <0.001 | Good |
| -Rantanen et al. (2008) | 0.14 | 0.03 | <0.001 | Good |
| -Vander Elst et al. (2026) | 0.16 | 0.03 | <0.001 | Good |
| -Neto et al. (2016) | 0.16 | 0.03 | <0.001 | Fair |
| -Zu et al. (2020b) | 0.15 | 0.03 | <0.001 | Fair |
| -Drummond et al. (2017) | 0.14 | 0.03 | <0.001 | Poor |
| -Sun et al. (2021) | 0.15 | 0.03 | <0.001 | Poor |
| -Zu et al. (2020a) | 0.15 | 0.03 | <0.001 | Poor |
| **Work-to-family conflict and insomnia symptoms** |  |  |  |  |
| Not sufficient for analysis (Study n=2) | - | - | - | - |
| **Family-to-work conflict and depressive symptoms** |  |  |  |  |
| All Studies | 0.11 | 0.03 | 0.003 |  |
| -Bergs et al. (2018) | 0.11 | 0.03 | 0.007 | Good |
| -Cho et al. (2021) | 0.08 | 0.02 | 0.006 | Good |
| -Coursolle et al. (2010) | 0.12 | 0.03 | 0.003 | Good |
| -Frone et al. (1997) | 0.10 | 0.03 | 0.006 | Good |
| -Kayaalp et al. (2021) | 0.11 | 0.03 | 0.012 | Good |
| -Yucel et al. (2022) | 0.12 | 0.03 | 0.008 | Good |
| **Family-to-work conflict and burnout** |  |  |  |  |
| All Studies | 0.06 | 0.03 | 0.086 |  |
| -Brzykcy et al. (2024) | 0.07 | 0.04 | 0.164 | Good |
| -Ren et al. (2025) | 0.08 | 0.02 | 0.013 | Fair |
| -Siu & Ng (2021) | 0.05 | 0.03 | 0.186 | Fair |
| -Innstrand et al. (2008) | 0.06 | 0.04 | 0.189 | Poor |
| -Langballe et al. (2011) | 0.04 | 0.03 | 0.206 | Poor |
| **Family-to-work conflict and general mental distress** |  |  |  |  |
| All Studies | 0.12 | 0.04 | 0.021 |  |
| -Matthews et al. (2014) | 0.13 | 0.05 | 0.054 | Good |
| -Rantanen et al. (2008) | 0.09 | 0.04 | 0.041 | Good |
| -Vander Elst et al. (2026) | 0.15 | 0.04 | 0.012 | Good |
| -Zu et al. (2020b) | 0.11 | 0.04 | 0.046 | Fair |
| -Drummond et al. (2017) | 0.14 | 0.05 | 0.033 | Poor |
| -Zu et al. (2020a) | 0.11 | 0.04 | 0.046 | Poor |

β **=** Standard deviation (SD) difference in the mental health outcome per 1-SD higher work–family conflict. SE = standard error.

| Table I. Sensitivity test without studies with poor quality | | | |
| --- | --- | --- | --- |
|  | **Pooled β** | **Pooled SE** | ***p*-value** |
| Work-to-family conflict and depressive symptoms |  |  |  |
| All Studies | 0.11 | 0.02 | <0.001 |
| Good and fair studies (Study n=9) | 0.11 | 0.03 | 0.001 |
| Work-to-family conflict and burnout |  |  |  |
| All Studies | 0.20 | 0.04 | <0.001 |
| Good and fair studies (Study n=5) | 0.12 | 0.03 | 0.001 |
| Work-to-family conflict and general mental well-being |  |  |  |
| All Studies | -0.10 | 0.01 | <0.001 |
| Good and fair studies (Study n=4) | -0.08 | 0.02 | 0.003 |
| Work-to-family conflict and general mental distress |  |  |  |
| All Studies | 0.15 | 0.03 | <0.001 |
| Good and fair studies (Study n=4) | 0.15 | 0.03 | <0.001 |
| Work-to-family conflict and insomnia symptoms |  |  |  |
| Not sufficient for analysis (Study n=1) | - | - | - |
| Family-to-work conflict and depressive symptoms |  |  |  |
| No poor-quality study existed | - | - | - |
| Family-to-work conflict and burnout |  |  |  |
| All Studies | 0.06 | 0.03 | 0.086 |
| Good and fair studies (Study n=3) | 0.03 | 0.04 | 0.476 |
| Family-to-work conflict and general mental distress |  |  |  |
| All Studies | 0.12 | 0.04 | 0.021 |
| Good and fair studies (Study n=3) | 0.13 | 0.06 | 0.077 |

β **=** Standard deviation (SD) difference in the mental health outcome per 1-SD higher work–family conflict. SE = standard error.

# **
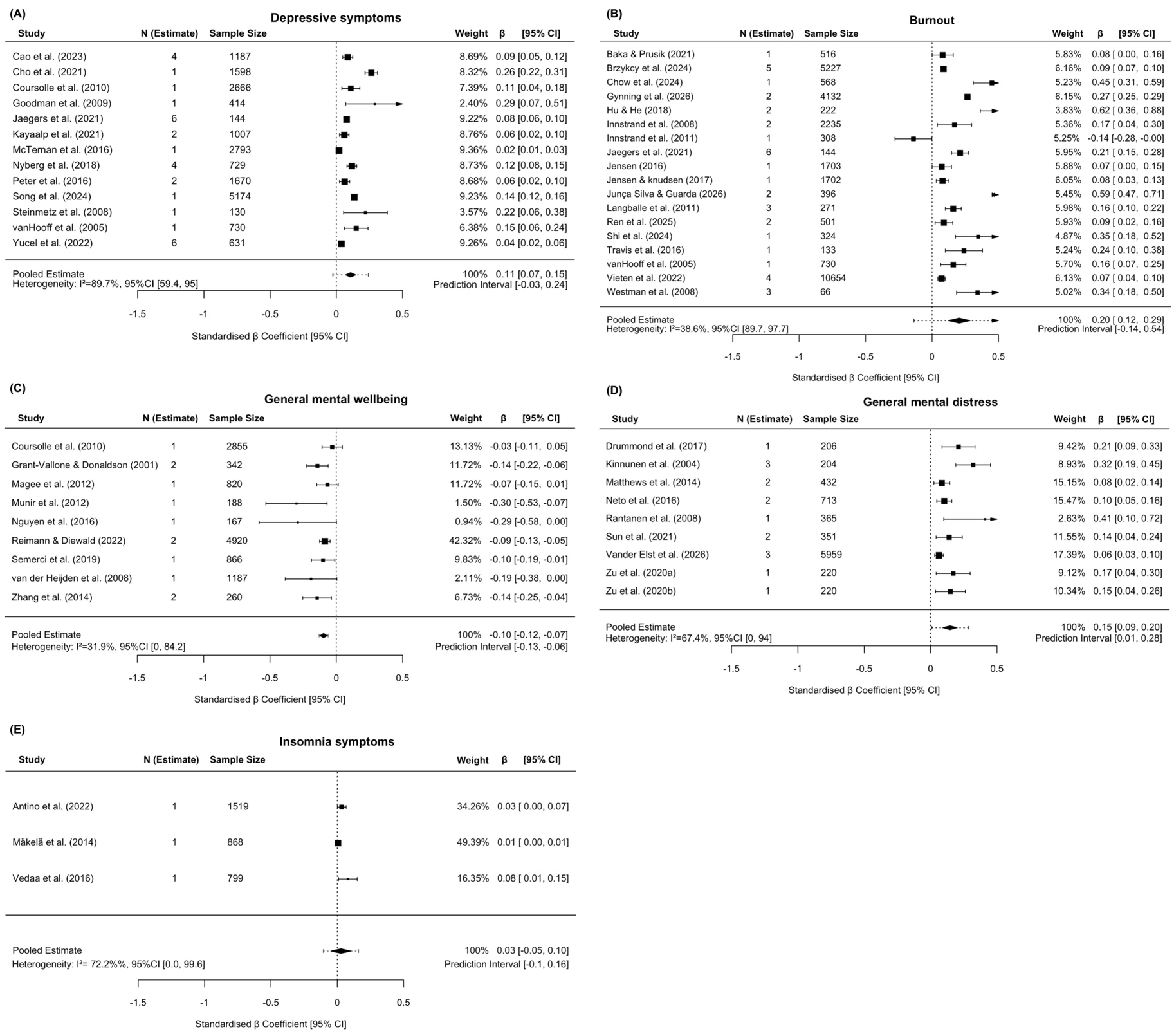
*Fig A*. Forest plots for the association between work-to-family conflict and (A) Depressive symptoms, (B) Burnout, (C) General mental wellbeing, (D) General mental distress, (E) Insomnia symptoms.**

**Notes:** Size of the squares indicates the weight of each individual effect size for the pooled effect. N(Estimate) = number of relevant effect sizes reported in the study. β **=** Standard deviation (SD) difference in the mental health outcome per 1-SD higher work–family conflict. Pooled overall effect sizes irrespective of covariates adjusted for in the analysis. Two-level meta-analysis was conducted for the association between work-to-family conflict and insomnia, three-level meta-analyses were conducted for other associations.

***Fig B.* Forest plot for the relationship between work-to-family conflict and depressive symptoms after adjusting for demographics, work-related factors, family-related factors, and baseline mental health.**
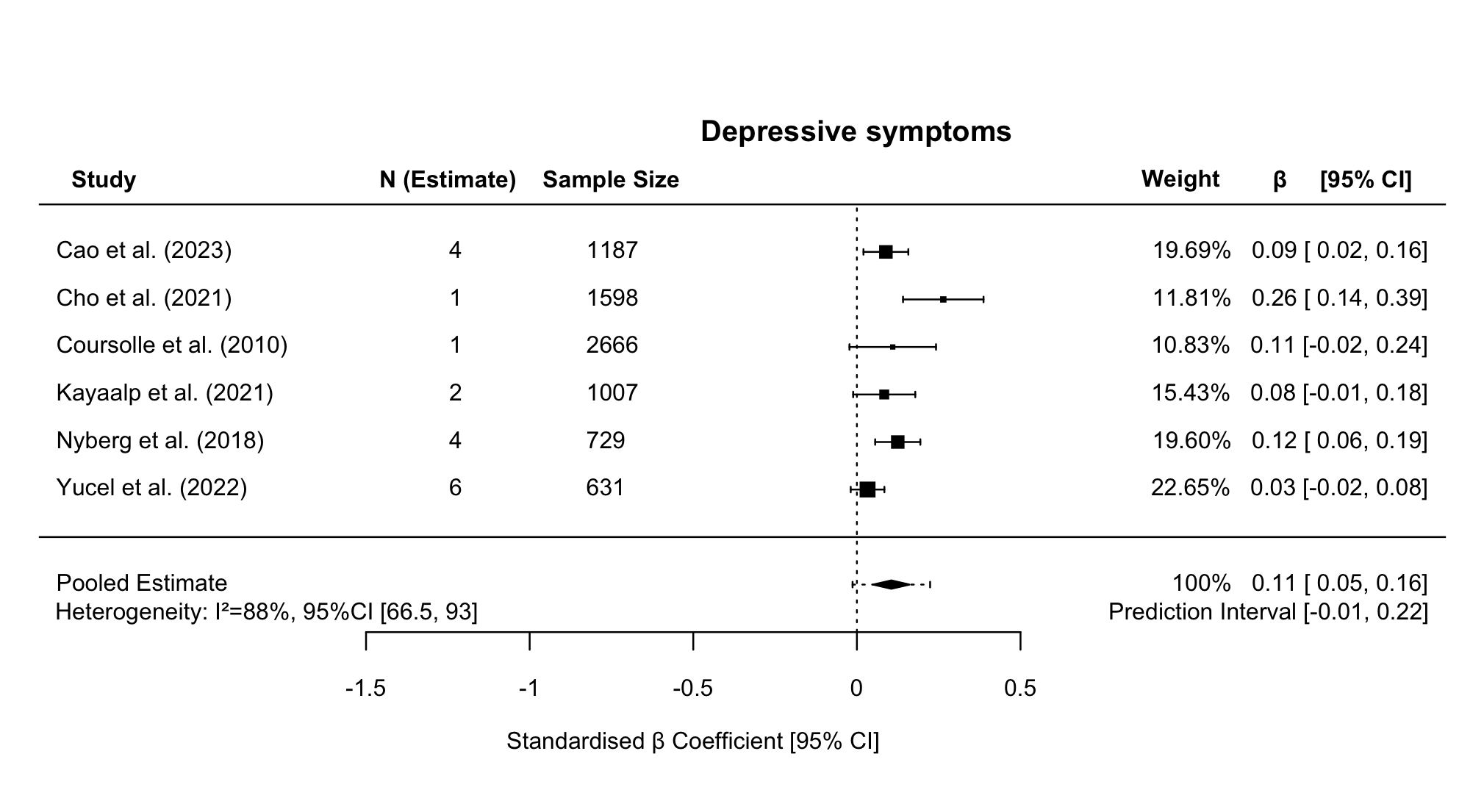


**Notes:** Size of the squares indicates the weight of each individual effect size for the pooled effect. N(Estimate) = number of relevant effect sizes reported in the study. β = Standard deviation (SD) difference in the mental health outcome per 1-SD higher work–family conflict.

# **
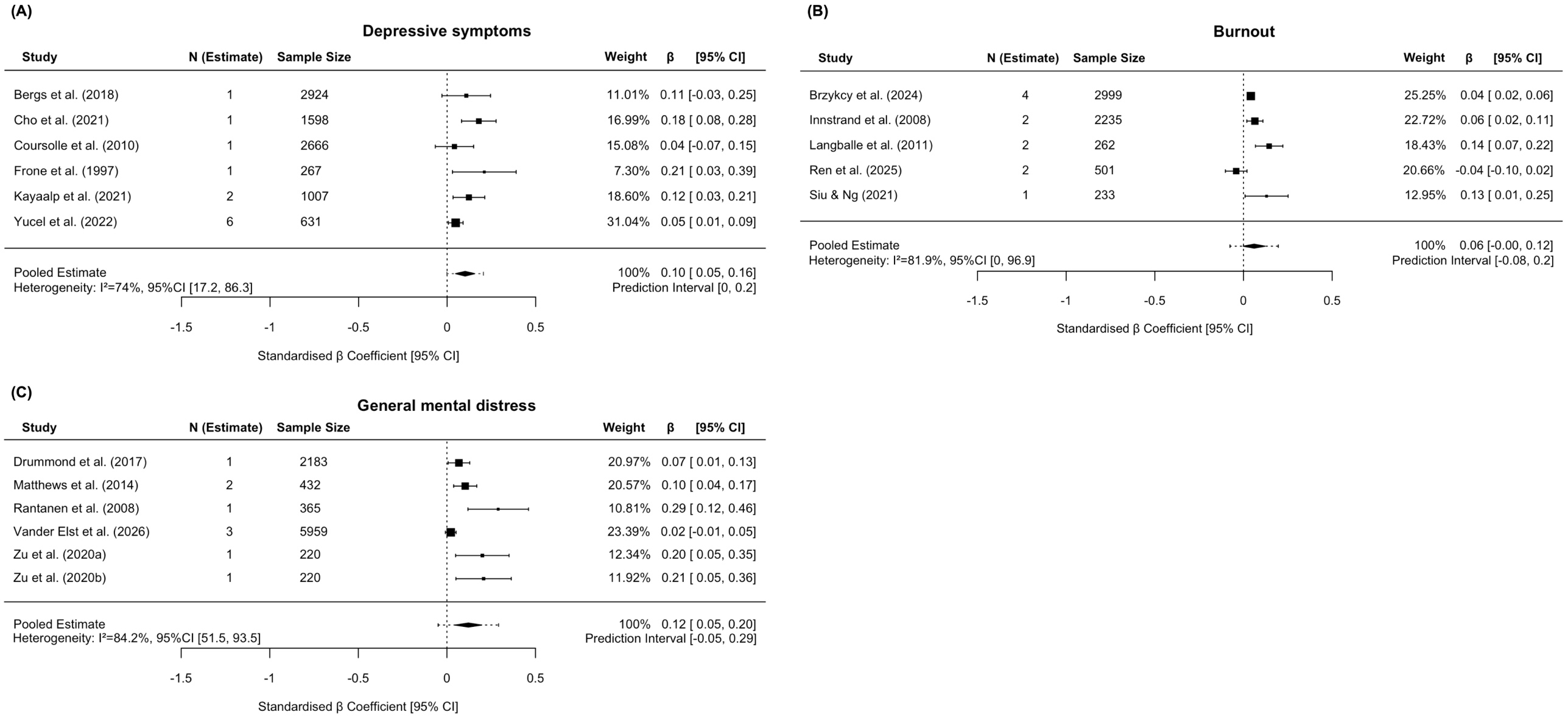
*Fig C.*** **Forest plots for the association between family-to-work conflict and (A) Depressive symptoms, (B) Burnout, (C) General mental distress.**

**Notes:** Size of the squares indicates the weight of each individual effect size for the pooled effect. N(Estimate) = number of relevant effect sizes reported in the study. β = Standard deviation (SD) difference in the mental health outcome per 1-SD higher work–family conflict. Pooled overall effect sizes irrespective of covariates adjusted for in the analysis.

#

# **
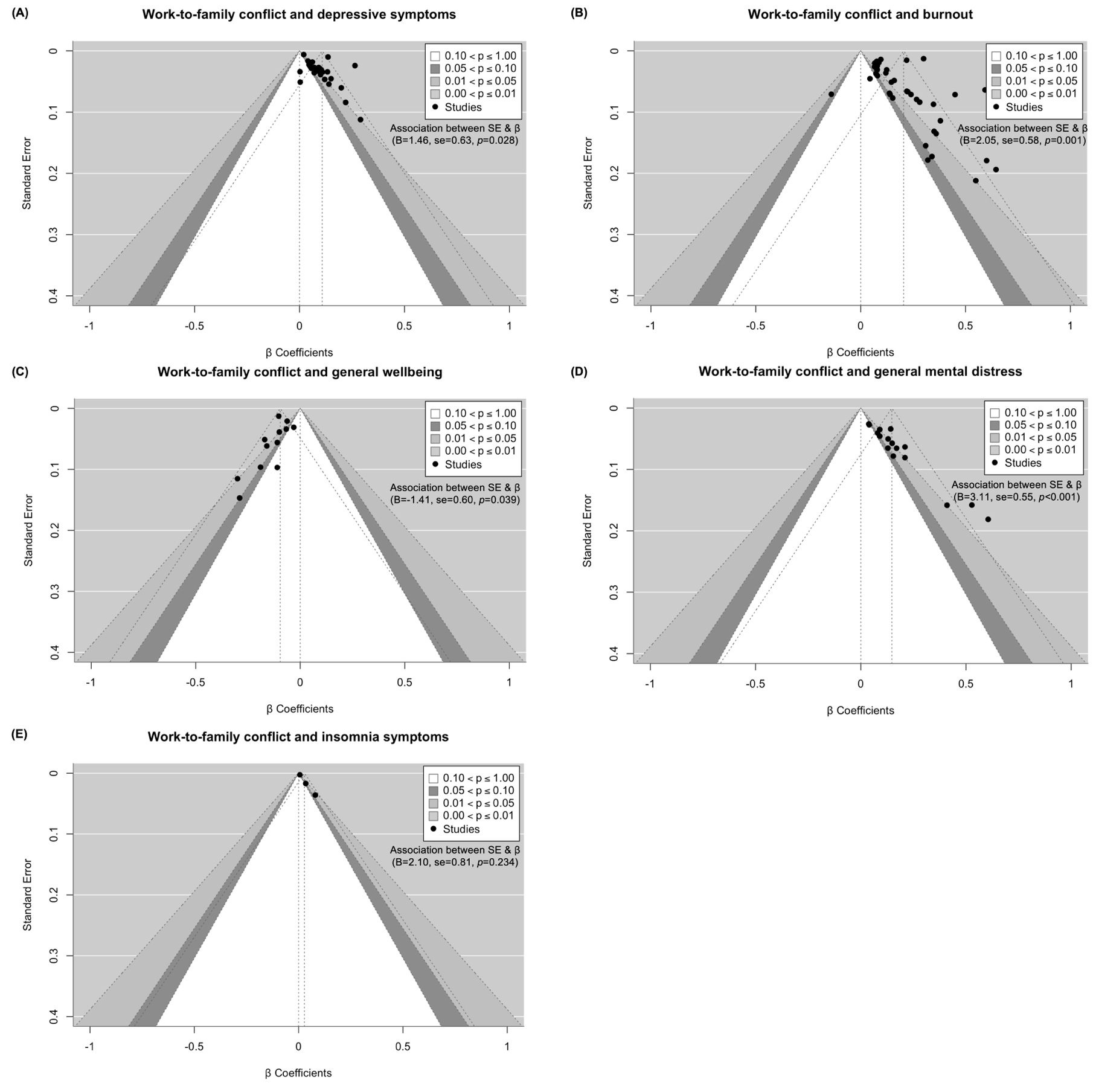
*Fig D.* Contour-enhanced funnel plot of publication bias for the relationship between work-to-family conflict and (A) Depressive symptoms, (B) Burnout, (C) General mental wellbeing, (D) General mental distress, (E) Insomnia symptoms.**

**Note:** SE = standard error. β = Standard deviation (SD) difference in the mental health outcome per 1-SD higher work–family conflict. B = point difference in β per 1-point higher SE.

# **
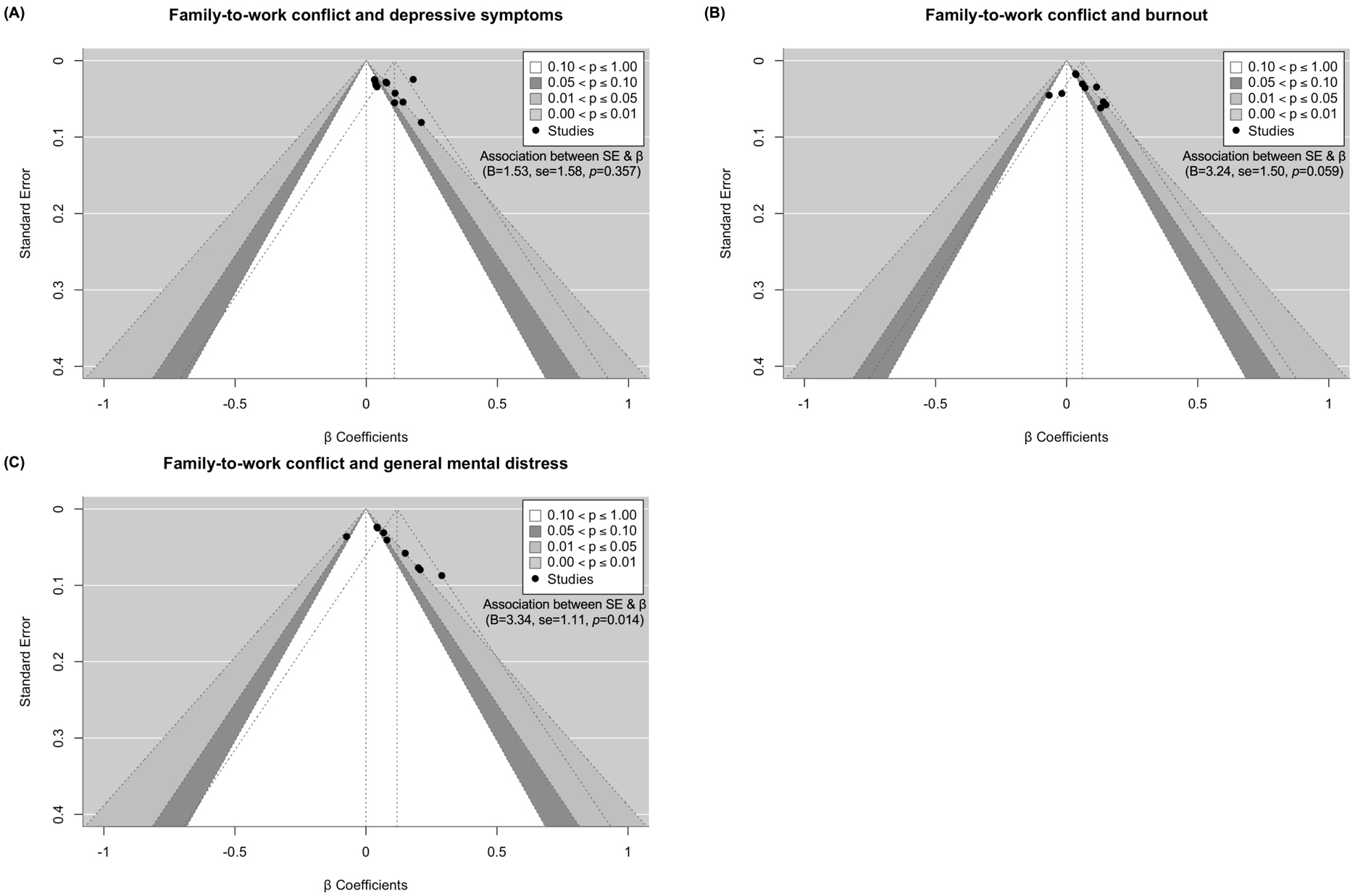
*Fig E.* Contour-enhanced funnel plot of publication bias for the relationship between family-to-work conflict and (A) Depressive symptoms, (B) Burnout, (C) General mental distress.**

**Note:** SE = standard error. β = Standard deviation (SD) difference in the mental health outcome per 1-SD higher work–family conflict. B = point difference in β per 1-point higher SE.

# **
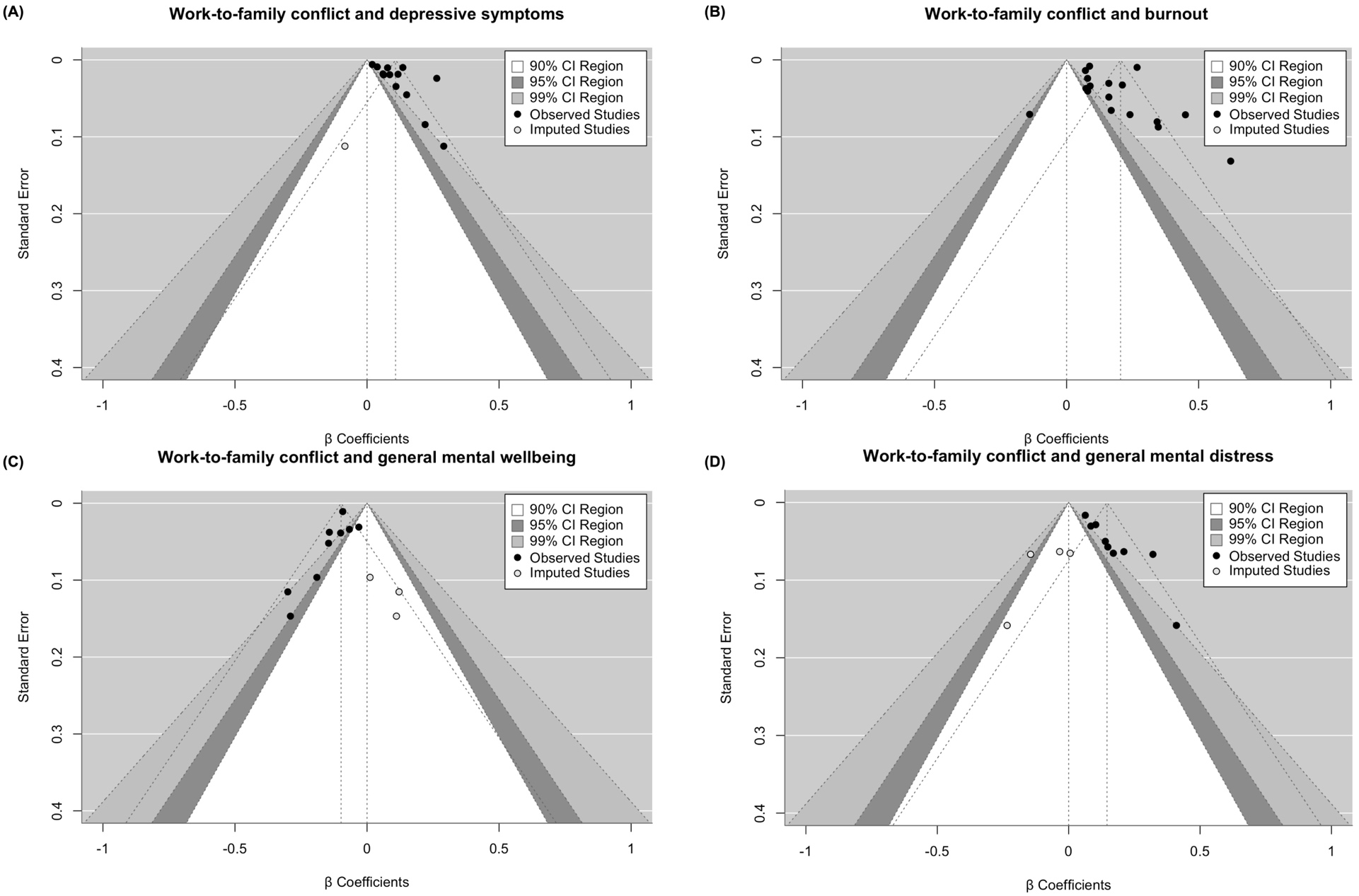
*Fig F.* Contour-enhanced funnel plot of publication bias for the relationship between work-to-family conflict and (A) Depressive symptoms, (B) Burnout, (C) General mental well-being, (D) General mental distress, after applying the trim-and-fill method at study-level.**

**Note:** β = Standard deviation (SD) difference in the mental health outcome per 1-SD higher work–family conflict.

#
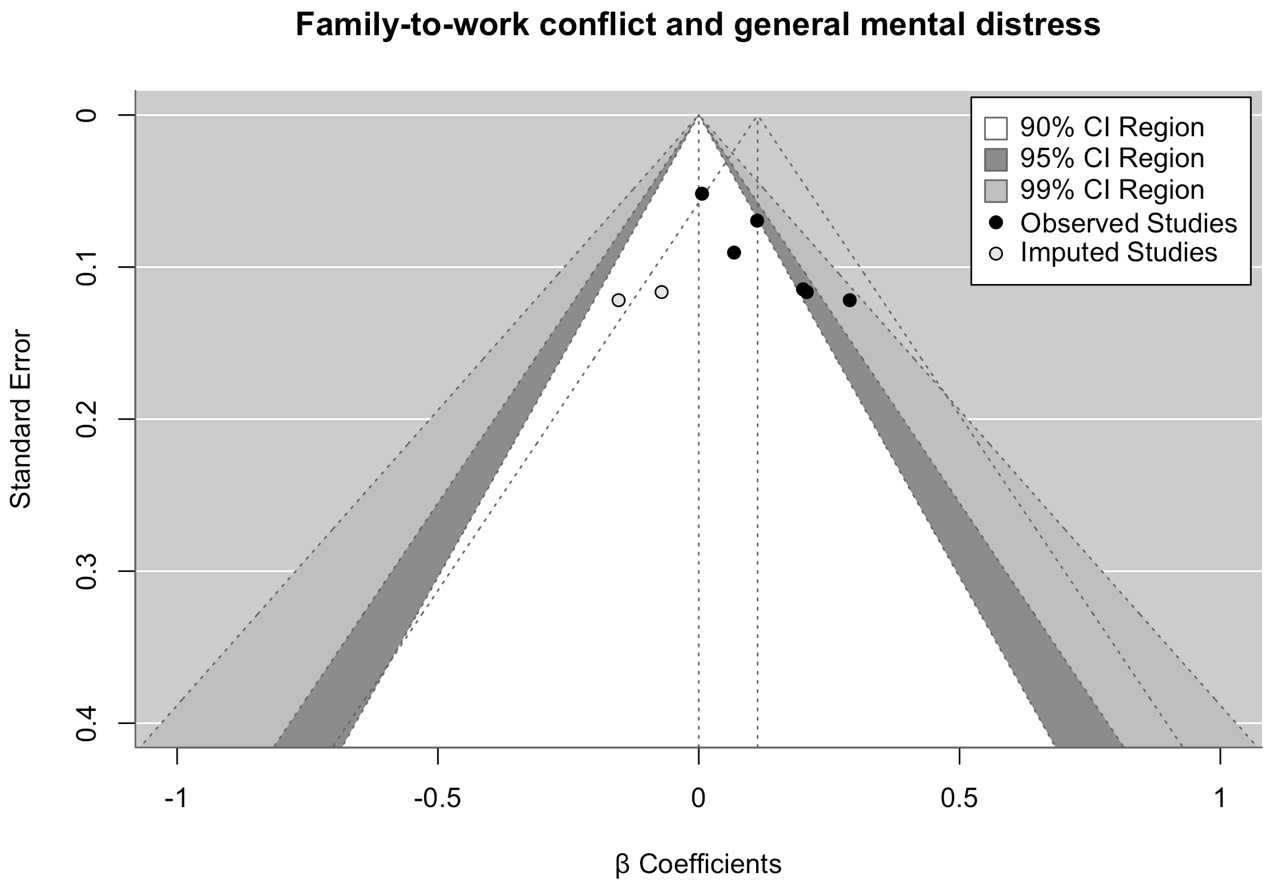
***Fig G.* Contour-enhanced funnel plot of publication bias for the relationship between family-to-work conflict and general mental distress, after applying the trim-and-fill method at study-level.**

**Note:** β = Standard deviation (SD) difference in the mental health outcome per 1-SD higher work–family conflict.

**
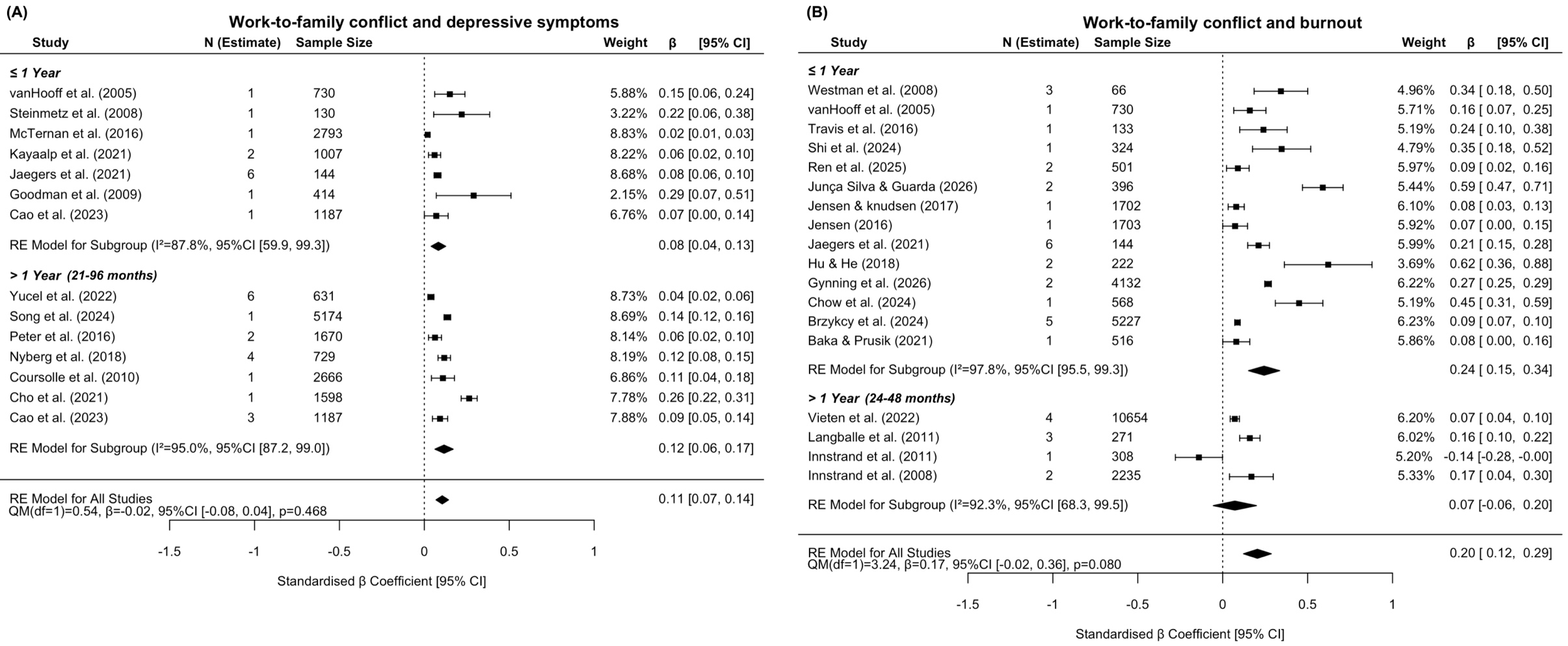
*****Fig H.* Work-to-family conflict and mental health by follow-up length within or over one year.** **(A) Work-to-family conflict and depressive symptoms, (B) Work-to-family conflict and burnout.**

**Note:** Size of the squares indicates the weight of each individual effect size for the pooled effect. N(Estimate) = number of relevant effect sizes reported in the study. β = Standard deviation (SD) difference in the mental health outcome per 1-SD higher work–family conflict. β for subgroup comparison indicates the mean difference in pooled effects between follow-up length subgroups, with follow-up length > 1 year as the reference group. Pooled effects from studies in all control conditions.

#
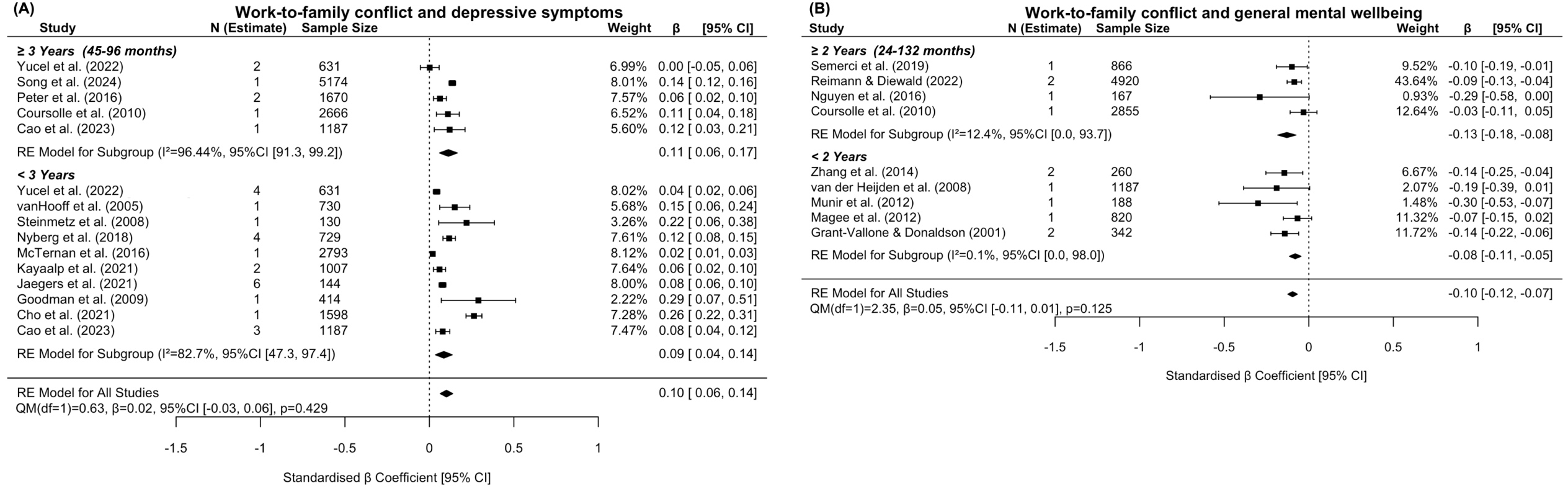
***Fig I.* Work-to-family conflict and mental health by follow-up length with cut-offs of two and three years. (A) Work-to-family conflict and depressive symptoms with a three-year cut-off, (B) Work-to-family conflict and general mental wellbeing with a two-year cut-off.**

**Note:** Size of the squares indicates the weight of each individual effect size for the pooled effect. N(Estimate) = number of relevant effect sizes reported in the study. β = Standard deviation (SD) difference in the mental health outcome per 1-SD higher work–family conflict. β for subgroup comparison indicates the mean difference in pooled effects between follow-up length subgroups, with the longer follow-up length as the reference group. Pooled effects from studies in all control conditions.

# **
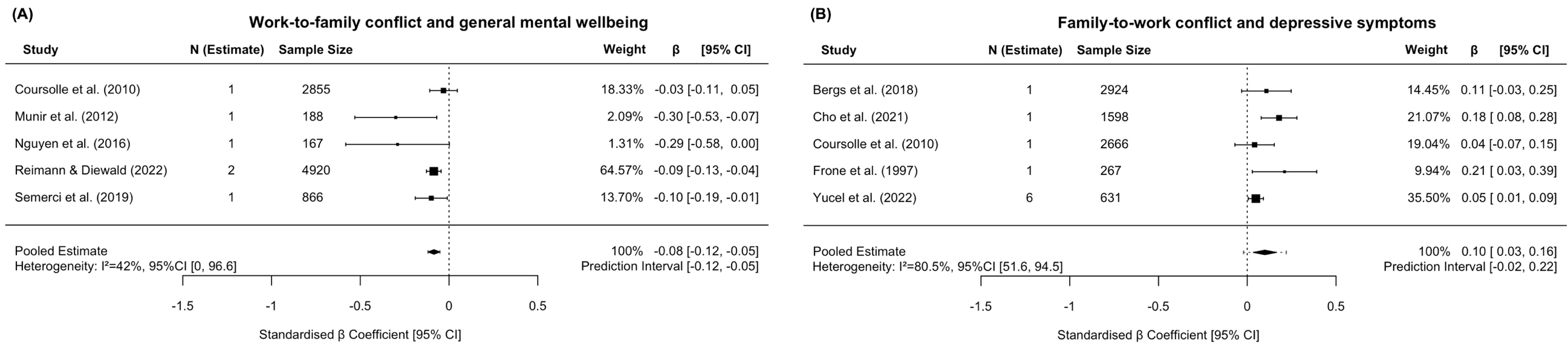
*Fig J.* Forest plot for the relationship between work-family conflict and mental health with follow-up interval over 12 months. (A) Work-to-family conflict and general mental wellbeing. (B) Family-to-work conflict and depressive symptoms.**

**Notes:** Size of the squares indicates the weight of each individual effect size for the pooled effect. N(Estimate) = number of relevant effect sizes reported in the study. β = Standard deviation (SD) difference in the mental health outcome per 1-SD higher work–family conflict. Pooled effects from studies in all control conditions.

# **
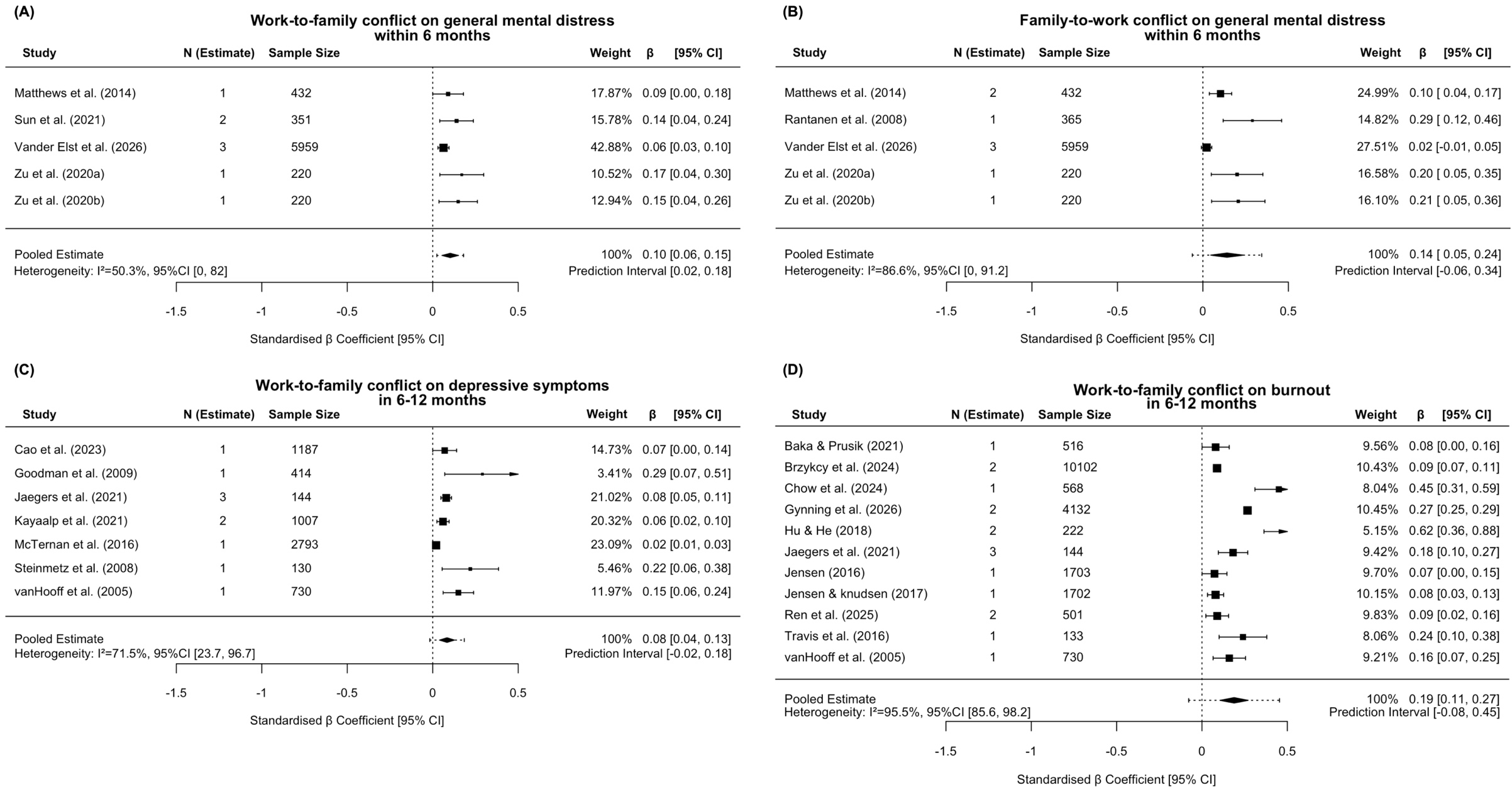
*Fig K.* Forest plot for the relationship between work-family conflict and mental health with follow-up interval within 12 months. (A) Work-to-family conflict and general mental distress (B) Family-to-work conflict and general mental distress. (C) Work-to-family conflict and depressive symptoms. (D) Work-to-family conflict and burnout.**

**Notes:** Size of the squares indicates the weight of each individual effect size for the pooled effect. N(Estimate) = number of relevant effect sizes reported in the study. β = Standard deviation (SD) difference in the mental health outcome per 1-SD higher work–family conflict. Pooled effects from studies in all control conditions. **(A)** Work-to-family conflict and general mental distress within 6 months. **(B)** Family-to-work conflict and general mental distress within 6 months. **(C)** Work-to-family conflict and depressive symptoms in 6-12 months. **(D)** Work-to-family conflict and burnout in 6-12 months

# ***Fig L.* Forest plots for the association between work-to-family conflict and mental health by geographical location after excluding US studies. (A) Burnout, (B) General mental distress.**


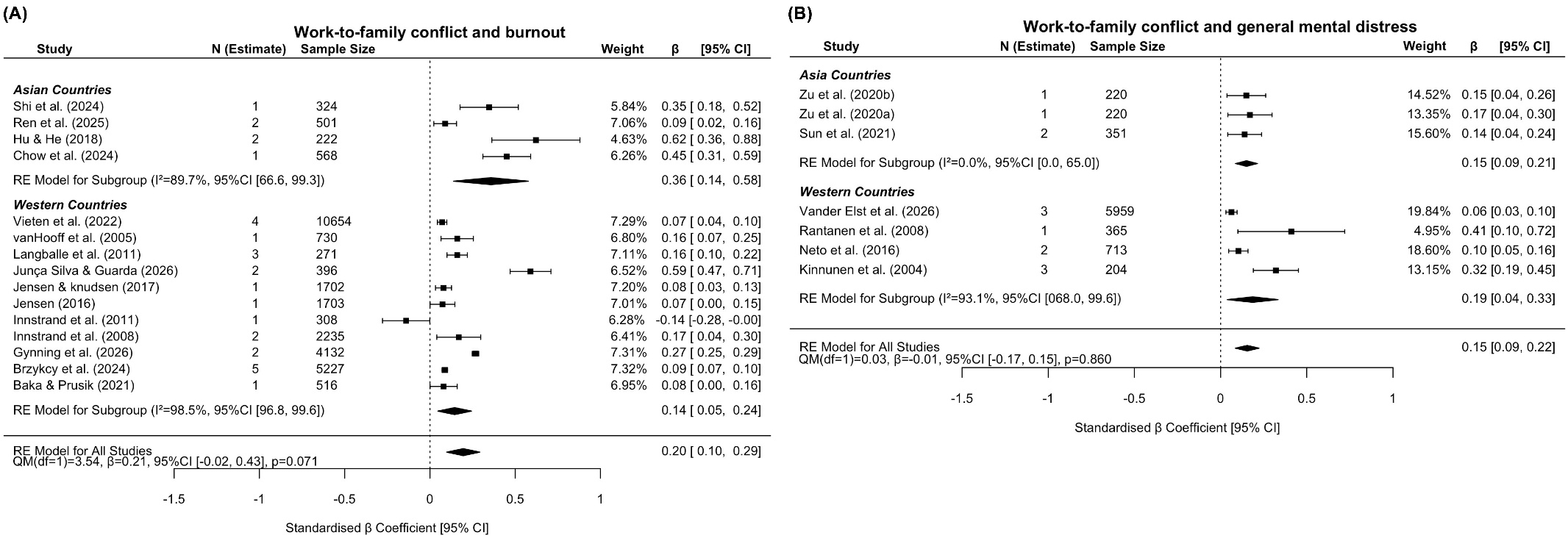


**Note:** N(Estimate) = number of relevant effect sizes reported in the study. β = Standard deviation (SD) difference in the mental health outcome per 1-SD higher work–family conflict. β for subgroup comparison indicates the mean difference in pooled effects between geographical location subgroups, with Western countries as the reference group. Available Asian studies were all from Chinese populations. Available Western studies are from Germany, Netherlands, Norway, Portugal, Sweden and Poland for burnout, and from Belgium, Finland, Portugal, and Finland for general mental distress. Pooled effects from studies in all control conditions.

**Reference**

1 Bring J. How to Standardize Regression Coefficients. *The American Statistician* 1994; **48**: 209–13.

2 Harrer M, Cuijpers P, Furukawa TA, Ebert DD. Effect Sizes. In: Doing Meta-Analysis in R. Boca Raton, FL and London: Chapmann & Hall/CRC Press, 2021. https://bookdown.org/MathiasHarrer/Doing_Meta_Analysis_in_R/effects.html (accessed March 10, 2024).

3 Baka Ł, Prusik M. Towards Better Understanding of the Harmful Impact of Hindrance and Challenge Stressors on Job Burnout of Nurses. A One-Year Cross-Lagged Study on Mediation Role of Work-Family Conflict. *Frontiers in Psychology* 2021; **12**: 696891.

4 Chow TS, Tang CS-K, Siu TSU, Kwok HSH. Examining the roles of self-compassion and self-control in managing work–family conflicts and preventing burnout during the pandemic: A three-wave longitudinal study in China. *International Journal of Psychology* 2024; **59**: 1015–25.

5 Costa A, Caldas de Almeida T, Fialho M, *et al.* Mental Health of Healthcare Professionals: Two Years of the COVID-19 Pandemic in Portugal. *International Journal of Environmental Research and Public Health* 2023; **20**: 3131.

6 Gynning BE, Christiansen F, Lidwall U, Brulin E. Impact of work–life interference on burnout and job discontent: A one-year follow-up study of physicians in Sweden. *Scand J Work Environ Health* 2024; **50**: 519–26.

7 Gynning BE, De Beer LT, Karlsson E, *et al.* Relative impact of psychosocial factors on burnout across healthcare professional roles. *Occup Med (Lond)* 2026; **76**: 21–8.

8 Hertzberg TK, Rø KI, Vaglum PJW, *et al.* Work-home interface stress: an important predictor of emotional exhaustion 15 years into a medical career. *Industrial Health* 2016; **54**: 139–48.

9 Hu Q, He Q. Longitudinal Study on the Effects of Work Engagement and Workaholism on Job Satisfaction and Emotional Exhaustion: The Mediating Role of Work-Family Conflict [In Chinese]. *Chinese Journal of Clinical Psychology* 2018; **26**: 1016–20.

10 Jaegers LA, Vaughn MG, Werth P, Matthieu MM, Ahmad SO, Barnidge E. Work–Family Conflict, Depression, and Burnout Among Jail Correctional Officers: A 1-Year Prospective Study. *Safety and Health at Work* 2021; **12**: 167–73.

11 Jensen MT. A two wave cross-lagged study of work-role conflict, work-family conflict and emotional exhaustion. *Scandinavian Journal of Psychology* 2016; **57**: 591–600.

12 Jensen MT, Knudsen K. A two-wave cross-lagged study of business travel, work–family conflict, emotional exhaustion, and psychological health complaints. *European Journal of Work and Organizational Psychology* 2017; **26**: 30–41.

13 Leineweber C, Baltzer M, Magnusson Hanson LL, Westerlund H. Work-family conflict and health in Swedish working women and men: a 2-year prospective analysis (the SLOSH study). *Eur J Public Health* 2013; **23**: 710–6.

14 Lizano EL, Mor Barak ME. Workplace demands and resources as antecedents of job burnout among public child welfare workers: A longitudinal study. *Children and Youth Services Review* 2012; **34**: 1769–76.

15 Richter A, Schraml K, Leineweber C. Work–family conflict, emotional exhaustion and performance-based self-esteem: reciprocal relationships. *International Archives of Occupational and Environmental Health* 2015; **88**: 103–12.

16 Shi G, Yan W, Pang H, Liu Z, Zheng X. Workplace fear of missing out and job burnout: The chain mediating effect of psychological detachment and work–family conflict. *Social Behavior and Personality: an international journal* 2024; **52**: 13008E-13016E.

17 Travis DJ, Lizano EL, Mor Barak ME. ‘I’m So Stressed!’: A Longitudinal Model of Stress, Burnout and Engagement among Social Workers in Child Welfare Settings. *The British Journal of Social Work* 2016; **46**: 1076–95.

18 van Hooff MLM, Geurts SAE, Taris TW, *et al.* Disentangling the causal relationships between work-home interference and employee health. *Scand J Work Environ Health* 2005; **31**: 15–29.

19 Vieten L, Wöhrmann AM, Michel A. Work-Time Control and Exhaustion: Internal Work-to-Home Interference and Internal Home-to-Work Interference as Mediators. *Int J Environ Res Public Health* 2022; **19**: 3487.

20 Westman M, Etzion D, Gattenio E. International business travels and the work-family interface: A longitudinal study. *Journal of Occupational and Organizational Psychology* 2008; **81**: 459–80.

21 Siu OL, Ng TK. Family-to-Work Interface and Workplace Injuries: The Mediating Roles of Burnout, Work Engagement, and Safety Violations. *International Journal of Environmental Research and Public Health* 2021; **18**: 11760.

22 Brzykcy AZ, Rönkkö M, Boehm SA, Goetz TM. Work–family conflict and strain: Revisiting theory, direction of causality, and longitudinal dynamism. *Journal of Applied Psychology* 2024; **109**: 1833–60.

23 Innstrand ST, Melbye Langballe E, Arild Espnes G, Falkum E, Gjerl⊘w Aasland O. Positive and negative work–family interaction and burnout: A longitudinal study of reciprocal relations. *Work & Stress* 2008; **22**: 1–15.

24 Innstrand ST, Langballe EM, Falkum E. The longitudinal effects of individual vulnerability, organisational factors, and work-home interaction on burnout among male church ministers in Norway. *Mental Health, Religion & Culture* 2011; **14**: 241–57.

25 Langballe EM, Innstrand ST, Aasland OG, Falkum E. The predictive value of individual factors, work-related factors, and work-home interaction on burnout in female and male physicians: A longitudinal study. *Stress and Health: Journal of the International Society for the Investigation of Stress* 2011; **27**: 73–85.

26 Lee Y, Eissenstat SJ. A longitudinal examination of the causes and effects of burnout based on the job demands-resources model. *International Journal for Educational and Vocational Guidance* 2018; **18**: 337–54.

27 Ren M, Han X, Xia Y, Ding D. Supportive coparenting in work–family conflict and parental burnout: Buffer or burden? *Family Relations* 2025; **75**: 301–22.

28 Cao H, Zhou N, Buehler C, Li X, Liang Y, Chen Y. Mothers’ work-to-family conflict, depressive symptoms, and parental role functioning: A five-wave, cross-lagged panel model from infancy through middle childhood. *Family Relations* 2023; **73**: 1178–200.

29 Cho E, Chen T-Y, Janke MC. A 2-Year Longitudinal Relationship Between Work-Family Conflict and Health Among Older Workers: Can Gardening Help? *J Appl Gerontol* 2021; **40**: 1330–41.

30 Goodman WB, Crouter AC, The Family Life Project Key Investigators. Longitudinal Associations between Maternal Work Stress, Negative Work-Family Spillover, and Depressive Symptoms. *Fam Relat* 2009; **58**: 245–58.

31 Hammer LB, Cullen JC, Neal MB, Sinclair RR, Shafiro MV. The longitudinal effects of work-family conflict and positive spillover on depressive symptoms among dual-earner couples. *J Occup Health Psychol* 2005; **10**: 138–54.

32 Magnusson Hanson LL, Leineweber C, Chungkham HS, Westerlund H. Work-home interference and its prospective relation to major depression and treatment with antidepressants. *Scand J Work Environ Health* 2014; **40**: 66–73.

33 Nyberg A, Peristera P, Bernhard-Oettel C, Leineweber C. Does work-personal life interference predict turnover among male and female managers, and do depressive symptoms mediate the association? A longitudinal study based on a Swedish cohort. *BMC Public Health* 2018; **18**: 828.

34 Song K, Lee M-A, Kim J. Double jeopardy: Exploring the moderating effect of educational mismatch in the relationship between work-family conflict and depressive symptoms among Korean working women. *Social Science & Medicine* 2024; **340**: 116501.

35 Steinmetz H, Frese M, Schmidt P. A longitudinal panel study on antecedents and outcomes of work–home interference. *Journal of Vocational Behavior* 2008; **73**: 231–41.

36 Suh C, Punnett L. Surface-acting emotional labor predicts depressive symptoms among health care workers over a 2-year prospective study. *International Archives of Occupational and Environmental Health* 2020; **94**: 367–75.

37 Thorup L, Sørensen CLB, Biering K. The Association between Work-Life Conflict and Mental Health—A Cohort Study. *Journal of Occupational & Environmental Medicine* 2025; **67**: 313–21.

38 Bergs Y, Hoofs H, Kant Ij, Slangen J, Jansen NW. Work-family conflict and depressive complaints among Dutch employees: examining reciprocal associations in a longitudinal study. *Scand J Work Environ Health* 2018; **44**: 69–79.

39 Ju YJ, Park E-C, Ju H-J, *et al.* The influence of family stress and conflict on depressive symptoms among working married women: A longitudinal study. *Health Care Women Int* 2018; **39**: 275–88.

40 Coursolle KM, Sweeney MM, Raymo JM, Ho J-H. The Association Between Retirement and Emotional Well-being: Does Prior Work–Family Conflict Matter? *The Journals of Gerontology Series B: Psychological Sciences and Social Sciences* 2010; **65B**: 609.

41 Frone MR, Russell M, Cooper ML. Relation of work–family conflict to health outcomes: A four-year longitudinal study of employed parents. *Journal of Occupational and Organizational Psychology* 1997; **70**: 325–35.

42 Grice MM, McGovern PM, Alexander BH, Ukestad L, Hellerstedt W. Balancing Work and Family After Childbirth: A Longitudinal Analysis. *Women’s Health Issues* 2011; **21**: 19–27.

43 Kayaalp A, Page KJ, Rospenda KM. Caregiver Burden, Work-Family Conflict, Family-Work Conflict, and Mental Health of Caregivers: A Mediational Longitudinal Study. *Work Stress* 2021; **35**: 217–40.

44 McTernan WP, Dollard MF, Tuckey MR, Vandenberg RJ. Enhanced Co-Worker Social Support in Isolated Work Groups and Its Mitigating Role on the Work-Family Conflict-Depression Loss Spiral. *Int J Environ Res Public Health* 2016; **13**: 382.

45 Peter R, March S, du Prel J-B. Are status inconsistency, work stress and work-family conflict associated with depressive symptoms? Testing prospective evidence in the lidA study. *Soc Sci Med* 2016; **151**: 100–9.

46 Yucel D, Borgmann L-S. Work-family conflict and depressive symptoms among dual-earner couples in Germany: A dyadic and longitudinal analysis. *Soc Sci Res* 2022; **104**: 102684.

47 Caines V, Treuren GJM. The importance of external social support for workplace-related stress as we grow older. *Australasian Journal on Ageing* 2023; **n/a**. DOI:10.1111/ajag.13252.

48 Kinnunen U, Geurts S, Mauno S. Work-to-family conflict and its relationship with satisfaction and well-being: a one-year longitudinal study on gender differences. *Work & Stress* 2004; **18**: 1–22.

49 Laine H, Saastamoinen P, Lahti J, Rahkonen O, Lahelma E. The associations between psychosocial working conditions and changes in common mental disorders: a follow-up study. *BMC Public Health* 2014; **14**: 588.

50 Marti AR, Degerud E, Sterud T. Onset of Work-Life Conflict Increases Risk of Subsequent Psychological Distress in the Norwegian Working Population. *International Journal of Environmental Research and Public Health* 2022; **19**: 13292.

51 Matthews LS, Conger RD, Wickrama KAS. Work-Family Conflict and Marital Quality: Mediating Processes. *Social Psychology Quarterly* 1996; **59**: 62–79.

52 Neto M, Carvalho VS, Chambel MJ, Manuel S, Pereira Miguel J, de Fátima Reis M. Work-Family Conflict and Employee Well-Being Over Time: The Loss Spiral Effect. *J Occup Environ Med* 2016; **58**: 429–35.

53 Oshio T, Inoue A, Tsutsumi A. Does work-to-family conflict really matter for health? Cross-sectional, prospective cohort and fixed-effects analyses. *Soc Sci Med* 2017; **175**: 36–42.

54 Oshio T, Inoue A, Tsutsumi A. Examining the mediating effect of work-to-family conflict on the associations between job stressors and employee psychological distress: a prospective cohort study. *BMJ Open* 2017; **7**: e015608.

55 Petrie K, Gayed A, Spittal MJ, Glozier N, Shand F, Harvey SB. Work-related factors and the risk of common mental disorder 1 year later: A prospective cohort study among junior doctors. *Aust N Z J Psychiatry* 2024; **58**: 227–37.

56 Drummond S, O’Driscoll MP, Brough P, *et al.* The relationship of social support with well-being outcomes via work–family conflict: Moderating effects of gender, dependants and nationality. *Human Relations* 2017; **70**: 544–65.

57 Matthews RA, Wayne JH, Ford MT. A work-family conflict/subjective well-being process model: a test of competing theories of longitudinal effects. *J Appl Psychol* 2014; **99**: 1173–87.

58 Rantanen J, Kinnunen U, Feldt T, Pulkkinen L. Work-family conflict and psychological well-being: Stability and cross-lagged relations within one- and six-year follow-ups. *Journal of Vocational Behavior* 2008; **73**: 37–51.

59 Vander Elst T, Vandenbroeck S, Boets I, Godderis L. Telecommuting and psychological distress: a cross-lagged study during the COVID-19 pandemic. *International Journal of Environmental Health Research* 2026; **36**: 1–11.

60 Sun Y, Chen Y, Zhang Y. The Relationship between Work Pressure and Psychological Well-being among Information Technology Professionals [In Chinese]. *Occupation and Health* 2021; **37**: 628–34.

61 Zu X, Wu Y, Song Y, Zhang Z. The Effect of Received Neighboring Behavior on General Health: The Mediating Role of Work-Family Conflict. *Asia Pac J Public Health* 2020; **32**: 250–7.

62 Zu X, Zhang Z, Wu Y, Zheng J. The Spillover Effects of Supportive Neighboring Behavior on Mental Health and Career Satisfaction: A Longitudinal Research on Chinese Low-Income Employees. *Psychol Res Behav Manag* 2020; **13**: 507–15.

63 Grant-Vallone EJ, Donaldson SI. Consequences of work-family conflict on employee well-being over time. *Work & Stress* 2001; **15**: 214–26.

64 Magee CA, Stefanic N, Caputi P, Iverson DC. The association between job demands/control and health in employed parents: The mediating role of work-to-family interference and enhancement. *Journal of Occupational Health Psychology* 2012; **17**: 196–205.

65 Munir F, Nielsen K, Garde AH, Albertsen K, Carneiro IG. Mediating the effects of work-life conflict between transformational leadership and health-care workers’ job satisfaction and psychological wellbeing. *J Nurs Manag* 2012; **20**: 512–21.

66 Nguyen H, Sawang S. Juggling or struggling? Work and family interface and its buffers among small business owners. *Entrepreneurship Research Journal* 2016; **6**: 207–46.

67 Boz Semerci A, Volery T. Longitudinal investigation on personality traits and mental health relationships: the mediating role of work-family interference and enhancement. *Current Issues in Personality Psychology* 2019; **7**: 173–88.

68 Van Der Heijden BIJM, Demerouti E, Bakker AB, Hasselhorn TNSG coordinated by H-M. Work-home interference among nurses: reciprocal relationships with job demands and health. *Journal of Advanced Nursing* 2008; **62**: 572–84.

69 Zhang X-C, Siu OL, Hu J, Zhang W. Relationships Between Bidirectional Work-Family Interactions and Psychological Well-Being. *Journal of Personnel Psychology* 2014; published online May 8. https://econtent.hogrefe.com/doi/10.1027/1866-5888/a000107 (accessed March 14, 2024).

70 Reimann M, Diewald M. Good mental health despite work-family conflict? The within-domain and cross-domain buffering potentials of family and work resources. *Journal of Family Research* 2022; **34**: 1126–50.

71 Magnusson Hanson LL, Chungkham HS, Åkerstedt T, Westerlund H. The Role of Sleep Disturbances in the Longitudinal Relationship Between Psychosocial Working Conditions, Measured by Work Demands and Support, and Depression. *Sleep* 2014; **37**: 1977–85.

72 Mäkelä L, Bergbom B, Tanskanen J, Kinnunen U. The relationship between international business travel and sleep problems via work-family conflict. *Career Development International* 2014; **19**: 794–812.

73 Antino M, Ruiz-Zorrilla P, Sanz-Vergel AI, Leon-Perez JM, Rodriguez-Muñoz A. The role of job insecurity and work-family conflict on mental health evolution during COVID-19 lockdown. *European Journal of Work and Organizational Psychology* 2022; **31**: 667–84.

74 Vedaa Ø, Krossbakken E, Grimsrud ID, *et al.* Prospective study of predictors and consequences of insomnia: personality, lifestyle, mental health, and work-related stressors. *Sleep Med* 2016; **20**: 51–8.

75 Vleeshouwers J, Knardahl S, Christensen JO. A prospective study of work–private life conflict and number of pain sites: moderated mediation by sleep problems and support. *Journal of Behavioral Medicine* 2018; **42**: 234–45.

76 Maekubo K, Deguchi Y, Iwasaki S, *et al.* Associations between occupational stress, work–family imbalance, and harmful alcohol consumption among workers: A longitudinal study. *Psychiatry and Clinical Neurosciences Reports* 2025; **4**: e70177.

77 Wolff JM, Rospenda KM, Richman JA. Age Differences in the Longitudinal Relationship between Work-Family Conflict and Alcohol Use. *J Addict* 2014; **2014**: 354767.

78 Kelloway EK, Gottlieb BH, Barham L. The source, nature, and direction of work and family conflict: a longitudinal investigation. *J Occup Health Psychol* 1999; **4**: 337–46.

79 Smoktunowicz E, Cieślak R. How job and family demands impact change in perceived stress: A dyadic study. *Int J Occup Med Environ Health* 2018; **31**: 199–215.

80 Weale V, Lambert KA, Graham M, Stuckey R, Oakman J. Do work–family conflict or family–work conflict mediate relationships between work-related hazards and stress and pain? *American Journal of Industrial Medicine* 2023; **66**: 780–93.

81 Kinnunen U, Feldt T, Mauno S, Rantanen J. Interface between work and family: A longitudinal individual and crossover perspective. *Journal of Occupational and Organizational Psychology* 2010; **83**: 119–37.

82 Wang J, Patten SB, Currie S, Sareen J, Schmitz N. A population-based longitudinal study on work environmental factors and the risk of major depressive disorder. *Am J Epidemiol* 2012; **176**: 52–9.

83 Wang JL, Patten SB, Currie S, Sareen J, Schmitz N. Predictors of 1-year outcomes of major depressive disorder among individuals with a lifetime diagnosis: a population-based study. *Psychol Med* 2012; **42**: 327–34.

84 Razavi T, Clark C, Stansfeld SA. Work-family conflict as a predictor of common mental disorders in the 1958 British birth cohort. *Longitudinal and Life Course Studies* 2015; **6**: 264–78.
