# Supplementary material for: Work-family conflict and mental health: A systematic review and meta-analysis
Source: PLoS Med. 2026 Jul 17;23(7):e1005162. doi: 10.1371/journal.pmed.1005162 (PMC13379121; doi:10.1371/journal.pmed.1005162)
Supplement: S3 Checklist — (DOCX) [file pmed.1005162.s004.docx]

| **Meta-analysis of Observational Studies in Epidemiology Checklist** | |
| --- | --- |
| **Checklist Item** | **Reported on page** |
| **Reporting of background** | |
| Problem definition | Introduction section paragraph 1-5 |
| Hypothesis statement | Introduction section paragraph 6 |
| Description of study outcome(s) | Search Strategy and Eligibility Screening subsections under Methods section |
| Type of exposure or intervention used | Search Strategy and Eligibility Screening subsections under Methods section |
| Type of study designs used | Search Strategy and Eligibility Screening subsections under Methods section |
| Study population | Search Strategy and Eligibility Screening subsections under Methods section |
| **Reporting of search strategy** | |
| Qualifications of searchers (eg, librarians and investigators) | Eligibility Screening and Data Extraction subsections under Methods section |
| Search strategy, including time period included in the synthesis and keywords | Search Strategy and Eligibility Screening subsections under Methods section |
| Effort to include all available studies, including contact with authors | Search Strategy subsection under Methods section |
| Databases and registries searched | Search Strategy subsection under Methods section |
| Search software used, name and version, including special features used (eg, explosion) | Search Strategy and paragraph 1 of Statistical Analysis subsections under Methods section |
| Use of hand searching (eg, reference lists of obtained articles) | Search Strategy subsection under Methods section |
| List of citations located and those excluded, including justification | Study Characteristics subsection under Methods section, Supplementary Table S8, Supplementary reference list |
| Method of addressing articles published in languages other than English | Search Strategy subsection under Methods section |
| Method of handling abstracts and unpublished studies | Search Strategy subsection under Methods section |
| Description of any contact with authors | - |
| **Reporting of methods** | |
| Description of relevance or appropriateness of studies assembled for assessing the hypothesis to be tested | Study Characteristics subsection under Methods section, Supplementary Table S6, S7, S8, S9 |
| Rationale for the selection and coding of data (eg, sound clinical principles or convenience) | Data Extraction subsection and paragraph 2 of Statistical Analysis subsection of Methods section, Supplementary Table S5 |
| Documentation of how data were classified and coded (eg, multiple raters, blinding, and interrater reliability) | Data Extraction subsections under Methods section |
| Assessment of confounding (eg, comparability of cases and controls in studies where appropriate) | Statistical Analysis subsection of Methods section |
| Assessment of study quality, including blinding of quality assessors; stratification or regression on possible predictors of study results | Risk of Bias Detection subsection of Methods section, Supplementary Table S9 |
| Assessment of heterogeneity | Statistical Analysis subsection of Methods section, paragraph 3. |
| Description of statistical methods (e.g. complete description of fixed or random effects models, justification of whether the chosen models account for predictors of study results, dose-response models, or cumulative meta-analysis) in sufficient detail to be replicated | Statistical Analysis subsection of Methods section. |
| Provision of appropriate tables and graphics | Figure 1-5, Supplementary Table S10, S11, S12, Supplementary Figure S1-S12 |
| **Reporting of results** | |
| Graphic summarizing individual study estimates and overall estimate | Figure 1-5, Supplementary Figure S1-S3, Figure S8-S12 |
| Table giving descriptive information for each study included | Supplementary Table S7, S8 |
| Results of sensitivity testing (eg, subgroup analysis) | Page 20-23, Supplementary Table S10, S11, S12 |
| Indication of statistical uncertainty of findings | Overall Meta-analytic Effects and Subgroup Comparison subsections under Results section. |
| **Reporting of discussion** | |
| Quantitative assessment of bias (e.g., publication bias) | Sensitivity Analysis subsection under Results section, paragraph 2. |
| Justification for exclusion (e.g., exclusion of non-English-language citations) | Figure 1 |
| Assessment of quality of included studies | Quality Assessment subsection under Results section, Supplementary Table S9 |
| **Reporting of conclusions** | |
| Consideration of alternative explanations for observed results | Discussion section, paragraph 1,2,4. |
| Generalization of the conclusions (i.e., appropriate for the data presented and within the domain of the literature review) | Discussion section, paragraph 1,2,4,6. |
| Guidelines for future research | Discussion section, paragraph 3,4,5 |
| Disclosure of funding source | Financial Disclosure Statement section |
